# Between-Visit Asthma Symptom Monitoring With a Scalable Digital Intervention: A Randomized Clinical Trial

**DOI:** 10.1001/jamanetworkopen.2025.6219

**Published:** 2025-04-23

**Authors:** Robert S. Rudin, Savanna Plombon, Jorge Sulca Flores, Jessica L. Sousa, Jorge Rodriguez, Dinah Foer, Stuart Lipsitz, Maria O. Edelen, David W. Bates, Adriana Arcia, Anuj K. Dalal

**Affiliations:** 1 Health Care Division, RAND, Boston, Massachusetts; 2 Division of General Internal Medicine and Primary Care, Brigham and Women’s Hospital, Boston, Massachusetts; 3 Division of Allergy and Clinical Immunology, Brigham and Women’s Hospital, Boston, Massachusetts; 4 Harvard Medical School, Boston, Massachusetts; 5 Hahn School of Nursing and Health Science, University of San Diego, San Diego, California

## Abstract

**Importance:**

Asthma affects an estimated 7.7% of the US population and 262 million people worldwide. Symptom monitoring has demonstrated benefits but has not achieved widespread use.

**Objective:**

To assess the effect of a scalable asthma symptom monitoring intervention on asthma outcomes.

**Design, Setting, and Participants:**

This randomized clinical trial was conducted between July 2020 and March 2023 at 7 primary care clinics affiliated with an academic medical center (Brigham and Women’s Hospital in Boston, Massachusetts). Candidate patients with a diagnosis of asthma over a 20-month recruitment period (July 2020 to March 2022) were identified and categorized into tiers of varying disease activity based on electronic health record data. Eligible patients were adults (aged ≥18 years) and had a primary care practitioner in 1 of the 7 participating clinics.

**Intervention:**

Intervention group patients were asked to use a mobile health app to complete weekly symptom questionnaires; track notes, peak flows, and triggers; and view educational information. Patients who reported worsening or severe symptoms were offered clinical callback requests. App data were available in the electronic health record. Usual care group patients received general asthma guidance.

**Main Outcomes and Measures:**

The primary outcome was the mean change in Mini Asthma Quality of Life Questionnaire (MiniAQLQ) score for the intended 12-month study period. A change of 0.5 on a scale of 1 to 7 was considered a minimally important change. The secondary outcome was the mean number of asthma-related health care utilization events (urgent care visits, emergency department visits, or hospitalizations). Mean differences for all outcomes between groups were compared using robust linear regression models (generalized estimating equations) with treatment group as the only covariate.

**Results:**

Baseline questionnaires were completed by 413 patients (mean [SD] age, 52.2 [15.4] years; 321 women [77.7%]). Of these, 366 patients completed final questionnaires and were included in the primary analysis. MiniAQLQ scores increased 0.34 (95% CI, 0.19-0.49) in the intervention group and 0.11 (95% CI, −0.11 to 0.33) in the usual care group from baseline to final questionnaire completion (adjusted difference-in-difference, 0.23 [95% CI, 0.06-0.40]; *P* = .01); although the difference was statistically significant, it did not reach the threshold for a minimally important change. Intervention subgroups showed positive differences in MiniAQLQ scores relative to the usual care group, with noteworthy increases among individuals aged 18 to 44 years (adjusted difference-in-difference, 0.40 [95% CI, 0.13-0.66]), those with low baseline patient activation (adjusted difference-in-difference, 0.77 [95% CI, 0.30-1.24]), those with a low baseline MiniAQLQ score (adjusted difference-in-difference, 0.33 [95% CI, 0.07-0.59]), and those with uncontrolled asthma at baseline (adjusted difference-in-difference, 0.30 [95% CI, 0.05-0.54]). The intervention group had a mean of 0.59 (95% CI, 0.42-0.77) nonroutine asthma-related utilization events compared with 0.76 (95% CI, 0.55-0.96) in the usual care group (adjusted effect size, −0.16 [95% CI, −0.42 to 0.17]; *P* = .23).

**Conclusions and Relevance:**

In this randomized clinical trial of a scalable symptom monitoring intervention, the increase in asthma-related quality of life did not reach the threshold for a minimally important change. Exploratory analyses suggest possible benefits for patients with low levels of activation.

**Trial Registration:**

ClinicalTrials.gov Identifier: NCT04401332

## Key Points

**Question:**

Does a scalable asthma symptom monitoring intervention improve asthma-related quality of life?

**Findings:**

In this randomized clinical trial involving 413 patients across 7 primary care clinics, a mobile health–based intervention using weekly questionnaires led to a statistically significant but not clinically meaningful increase in patient-reported asthma-related quality of life. There was no change in nonroutine asthma-related health care utilization, although patients with low levels of self-assessed activation (defined by knowledge, skills, and confidence in managing their health) may derive perceptible benefit.

**Meaning:**

These findings did not reach the threshold for a minimally important change in asthma-related quality of life with a scalable asthma symptom monitoring intervention; however, subgroups of patients, particularly those with low levels of self-assessed activation, may experience benefit.

## Teaser

This randomized clinical trial evaluates the effect of a scalable digital intervention for between-visit symptom monitoring on asthma outcomes among adults.

## Introduction

Asthma affects an estimated 7.7% of the US population and 262 million people worldwide.^1,2^ For many of these individuals, asthma causes major impairments in quality of life and substantial planned and unplanned health care utilization, with disproportionately higher effects among minoritized populations and low-income populations.^3,4,5^

Routine monitoring of asthma symptoms is recommended by clinical guidelines to improve asthma control.^6,7,8^ Symptoms may be monitored at the time of a health care visit or between visits, with the benefits of between-visit symptom monitoring previously demonstrated in many research studies.^9^ These include observational studies^10^ as well as trials evaluating asthma interventions unrelated to symptom monitoring, such as medication regimens, in which improvements in asthma indicators are shown in the control groups that receive regular symptom questionnaires.^11^

Randomized trials of digital asthma health interventions have provided evidence that symptom monitoring improves outcomes for patients with asthma.^12,13^ However, prior trials have bundled together symptom monitoring with other, less scalable components,^9,14^ such as devices that track inhaler use,^15^ education delivered by clinicians,^12,13^ and personalized asthma action plans that require clinician input.^16^ While these other interventions hold promise to improve asthma outcomes, they are challenging to fully implement and sustain and may not be required to realize the benefits of symptom monitoring, which can be implemented at scale by leveraging the already widespread adoption of electronic health records (EHRs), patient portals, and smartphones.^17,18^ Understanding the benefits of between-visit symptom monitoring alone—as implemented in a scalable intervention—may foster more widespread adoption.

We sought to determine whether a between-visit symptom monitoring intervention specifically designed to scale (ie, implemented without other less scalable components) improved asthma outcomes. We designed and developed the intervention^18,19,20^ using the Nonadoption, Abandonment, Scale-up Spread, and Sustainability (NASSS) framework,^21,22^ which identifies domains along which an intervention can be assessed as ranging from simple to complex (ie, from more to less scalable). Our previous evaluations of the intervention’s implementation demonstrated promising scalability on NASSS domains, high adherence (72.3% of questionnaires completed), and high retention (78.9% of patients still using the app at 12 months).^23,24^ The aim of this analysis was to evaluate this intervention’s effectiveness on asthma-related quality of life and asthma-related health care utilization.

## Methods

### Study Design and Setting

We conducted a pragmatic randomized clinical trial (RCT), designed to inform clinical practice, of a clinically integrated asthma symptom monitoring intervention with 2 groups in 7 primary care clinics affiliated with a large academic health center, Brigham and Women’s Hospital, which is part of Mass General Brigham (MGB), in Boston, Massachusetts. The trial protocol and statistical analysis plan are provided in Supplement 1. The study was conducted between July 2020 and March 2023, beginning shortly after the beginning of the COVID-19 pandemic, and was entirely virtual except that some patients were recruited in person at the time of a visit with their primary care practitioner (PCP). Study protocols were approved by the institutional review boards at MGB and RAND. Participants provided written and verbal informed consent. The study followed the Consolidated Standards of Reporting Trials (CONSORT) reporting guideline.

### Patient Recruitment and Randomization

We identified candidate patients with a diagnosis of asthma over a 20-month recruitment period (July 2020 to March 2022) and categorized them into tiers of varying disease activity based on EHR data. Eligible patients were adults (aged ≥18 years) and had a PCP in 1 of the 7 participating clinics. Recruited patients were asked to complete a REDCap^25^ screening form to confirm eligibility to participate in the trial (eligibility criteria in Supplement 1). All recruitment materials were available in English and Spanish. Details of recruitment methodology have been previously published.^23^

After eligible patients had provided informed consent, those who completed baseline surveys were randomized in REDCap into intervention and control (hereinafter, usual care) groups using a block size of 4. Intervention group patients were asked to download and use the app on their smartphone. Usual care group patients received an email with general asthma advice (details are provided in eAppendix 1 in Supplement 2).

### Intervention

We designed a scalable intervention using principles of user-centered design; details of the intervention and design process have been published previously.^18,19,20^ Additional information about the intervention is available in eAppendix 1 in Supplement 2. Briefly, the intervention consisted of a patient-facing smartphone application (available in English and Spanish) that asked patients to complete a 5-item asthma symptom questionnaire each week,^19^ with the option to request a callback from a nurse for problematic symptoms (defined as a 3-point worsening compared with baseline or the prior week’s score, or severest symptom on any one question) as well as visit reminders and access to symptom graphs and educational materials, a PCP-facing EHR dashboard reflecting patient-entered data (with EHR inbox reminders sent before scheduled visits with intervention group patients), and a nurse-facing practice model (with EHR inbox messages sent to the appropriate nurse or nurse pool for patient callback requests, and a nurse-driven triage protocol available).

### Data Collection

Enrolled patients were asked to complete baseline, 6-month, and 12-month questionnaires in REDCap (eAppendix 2 in Supplement 2). Baseline questionnaires included the Asthma Control Measure (ACM), the Patient Activation Measure (PAM) (which assesses an individual’s knowledge, skills, and confidence for managing their health^26,27,28^ [hereinafter, activation]), the Short Health Literacy Survey (SLS), the Mini Asthma Quality of Life Questionnaire (MiniAQLQ), and a question about health care utilization due to asthma. The 6- and 12-month questionnaires included only the MiniAQLQ and health care utilization questions. The MiniAQLQ (used with permission) is a 15-item validated questionnaire available in English and Spanish.^29^ Demographic, clinical, and utilization data were collected from the health system EHR. Patients received $25 gift cards after they initially signed up and after they completed the 12-month questionnaire ($50 total).

### Measures

#### Outcomes

The primary outcome was change in mean 15-item MiniAQLQ score over the study period.^29^ A change of 0.5 on a scale of 1 to 7 was considered a minimally important change. The MiniAQLQ was sent to patients at baseline, 6 months, and 12 months via REDCap; patients ranged in terms of when they completed the questionnaires, with some completing them right away and others several weeks later. The secondary outcome was the numeric count of nonroutine asthma-related health care utilization events (defined as urgent care visits, emergency department visits, and hospitalizations with asthma as the primary diagnosis) that occurred at MGB-affiliated institutions during the 12-month study period, as reflected in data from the MGB EHR. Exploratory outcomes included change in the 4 MiniAQLQ subdomains (symptoms, activity limitations, emotional function, and environmental stimuli), asthma-related utilization in the outpatient setting (number of visits), and number of prednisone prescriptions over the 12-month study period.

#### Patient Characteristics

Baseline age, sex, race and ethnicity, marital status, primary language, education, socioeconomic status (median income by zip code), insurance status, study clinic, PCP type, patient portal status (activation status and use in the prior 6 months), smoking status (current, former, or never), asthma exacerbation events in the prior 12 months (≥1), environmental or seasonal allergy or allergic rhinitis comorbidity (based on problem list), Charlson Comorbidity Index score,^30^ and body mass index (BMI) were exacted from the MGB EHR. Baseline smartphone type, ACM score, PAM score, SLS score, and time between completion of baseline and final surveys were computed from REDCap data. Race and ethnicity was self-reported as Black, Hispanic, White, or other race or ethnicity (including American Indian or Alaska Native, Asian, and Native Hawaiian or Other Pacific Islander) or declined to answer; these data were collected because prior studies have shown associations between race and ethnicity and asthma outcomes.

### Statistical Analysis

Our primary analysis was the complete case intention-to-treat analysis recommended by Jakobsen et al^31^ and Groenwold et al,^32^ in which we did not impute missing values and used the time of final MiniAQLQ completion as a covariate. This approach is consistent with RCTs that measure quality of life outcomes,^33^ and our rates of missing data were comparable with prior, similar asthma studies.^12^ As recommended by White et al,^34^ we also performed a sensitivity analysis in which we used multiple imputation for all missing final MiniAQLQ scores. In the multiple imputation, we used the observed data for a patient (baseline and 6-month MiniAQLQ scores, as well as covariates sex, income, insurance, employment, and education) to impute the final MiniAQLQ scores. Our target was 500 patients based on estimated power of 90% or greater to detect a 0.5-point increase (defined as clinically significant) in mean MiniAQLQ score from baseline to study completion with a 2-sided type I error rate of 5% and an intracluster correlation coefficient of 0.01 for patients from the same physician. Our secondary analysis was intention to treat.

#### Outcome Models

We used robust linear regression models (generalized estimating equations) with treatment group as the only covariate and clustering by PCP to estimate mean differences between groups. We adjusted our outcomes for imbalances in baseline characteristics for participants who completed both the baseline and 12-month questionnaires. We adjusted models for sex, median income, education, insurance status, and employment because of differences in baseline characteristics. We also adjusted for time between completion of baseline and 12-month primary outcome questionnaires.

#### Sensitivity Analyses

We conducted 3 prespecified sensitivity analyses: (1) the primary analysis with multiple imputation of missing 12-month MiniAQLQ data, (2) the secondary analysis with the subset of patients who were included in the primary analysis, and (3) the secondary analysis with patient-reported data on asthma-related health care utilization (defined as urgent care visits, emergency department visits, and hospitalizations due to asthma symptoms), which included data from both inside and outside of the MGB health system.

#### Subgroup, Exploratory, and Post Hoc Analyses

We conducted analyses on the primary outcome (MiniAQLQ scores) for patients who completed both the baseline and final questionnaires according to the following subgroup characteristics: baseline age, sex, race and ethnicity, smoking status, PAM score (levels 3-4 for high or 1-2 for low), SLS score (14 of 15 or ≤13), MiniAQLQ score (above or below the median), and ACM score (controlled or uncontrolled). We also conducted the exploratory analyses to assess the effect of the intervention on the following: (1) mean change in patient responses for each of the 4 MiniAQLQ subdomains (the exposure to environmental stimuli domain includes being bothered by or avoiding dust, cigarette smoke, and weather or air pollution; the emotional function domain includes feeling frustrated because of asthma, afraid of not having asthma medication available, and concerned about having asthma; the symptoms domain includes shortness of breath, coughing, chest tightness and heaviness, difficulty sleeping, and wheezing; and the activity limitations domain includes limitations in strenuous, moderate, social, and work-related activities), (2) number of patients with asthma-related outpatient visits (defined as outpatient visits that occurred at MGB-affiliated locations during the study period, coded in EHR data with asthma as the primary diagnosis), (3) number of patients with 1 or more prednisone prescriptions during the study period (a proxy for asthma exacerbations), and (4) a repeated-measures analysis on the primary outcome to compare change in MiniAQLQ score during the first 6 months vs the second 6 months. We conducted post hoc analyses on the primary outcome to compare change in MiniAQLQ scores for patients with high and low adherence and retention defined by app questionnaire completion rates.

Statistical analyses were conducted using SAS, version 9.4 (SAS Institute Inc). We considered 2-sided *P* < .05 to be statistically significant for primary and secondary analyses.

## Results

### Sample Characteristics

**Figure 1.**
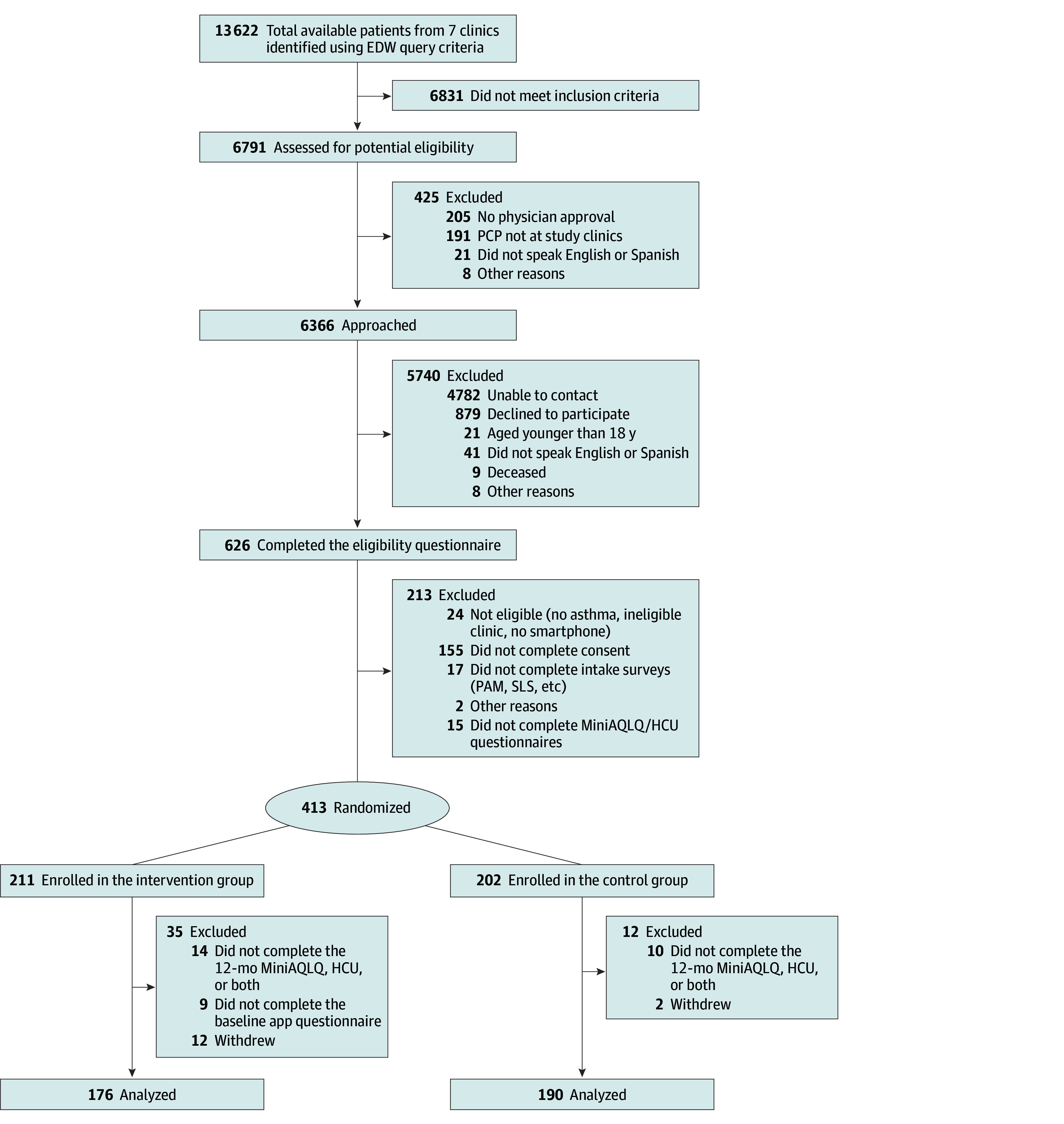
CONSORT Flow Diagram EDW indicates enterprise data warehouse; HCU, health care utilization; MiniAQLQ, Mini Asthma Quality of Life Questionnaire; PAM, Patient Activation Measure; PCP, primary care practitioner; SLS, Short Health Literacy Survey.

A total of 413 enrolled participants (mean [SD] age, 52.2 [15.4] years; 321 women [77.7%] and 92 [22.3%] men) completed all baseline questionnaires (Figure 1). Among these participants, 82 (19.9%) were Black, 77 (18.6%) were Hispanic, 226 (54.7%) were White, and 28 (6.8%) were of other race or ethnicity or declined to answer. There were 366 patients (88.6%) who completed the final questionnaire (176 in the intervention group and 190 in the usual care group). The remaining 47 patients (11.4%) were lost to follow-up (35 in the intervention group and 12 in the usual care group). Some differences in baseline characteristics were found among study groups (Table 1 and eTables 1 and 2 in Supplement 2). Compared with the usual care group, fewer patients in the intervention group were female (125 [71.0%] vs 155 [81.6%]), fewer were Black (28 [15.9%] vs 42 [22.1%]), more had a college degree (109 [61.9%] vs 97 [51.1%]), and more had commercial insurance (118 [67.1%] vs 106 [55.8%]). There were also some differences in baseline characteristics, including in age, sex, race and ethnicity, primary language, education, insurance status, and employment status, among the 47 patients lost to follow-up (eTable 1 in Supplement 2).

**Table 1.** Participant Characteristics at Baseline^a^

Characteristic	Enrolled patients (N = 413)	Intervention group (n = 211)	Usual care group (n = 202)	Completed 12-mo MiniAQLQ	
				Intervention group (n = 176)	Usual care group (n = 190)
Age, mean (SD), y	52.2 (15.4)	51.9 (15.5)	52.6 (15.4)	52.4 (15.3)	52.1 (15.5)
Sex					
Female	321 (77.7)	157 (74.4)	164 (81.2)	125 (71.0)	155 (81.6)
Male	92 (22.3)	54 (25.6)	38 (18.8)	51 (29.0)	35 (18.4)
Race and ethnicity					
Black	82 (19.9)	37 (17.5)	45 (22.3)	28 (15.9)	42 (22.1)
Hispanic	77 (18.6)	42 (19.9)	35 (17.3)	29 (16.5)	32 (16.8)
White	226 (54.7)	116 (55.0)	110 (54.5)	106 (60.2)	105 (55.3)
Other or missing^b^	28 (6.8)	16 (7.6)	12 (5.9)	13 (7.4)	11 (5.8)
Marital status					
Partnered	174 (42.1)	98 (46.5)	76 (37.6)	86 (48.9)	72 (37.9)
Single	236 (57.1)	111 (52.6)	125 (61.9)	88 (50.0)	117 (61.6)
Missing	3 (0.7)	2 (1.0)	1 (0.5)	2 (1.1)	1 (0.5)
Primary language English	394 (95.4)	202 (95.7)	192 (95.0)	169 (96.0)	183 (96.3)
Educational attainment					
Completed 8th grade or some high school but did not graduate	27 (6.5)	14 (6.6)	13 (6.4)	10 (5.7)	10 (5.3)
Graduated high school or obtained GED	118 (28.5)	56 (26.5)	62 (30.7)	40 (22.7)	58 (30.5)
Graduated college	220 (53.3)	118 (55.9)	102 (50.5)	109 (61.9)	97 (51.1)
Missing	48 (11.6)	23 (10.9)	25 (12.4)	17 (9.7)	25 (13.2)
Socioeconomic status, median income by zip code, $					
≤76 585	106 (25.7)	54 (25.6)	52 (25.7)	39 (22.2)	51 (26.8)
76 586-108 824	103 (24.9)	50 (23.7)	53 (26.2)	42 (23.9)	49 (25.8)
108 825-137 102	102 (24.7)	53 (25.1)	49 (24.3)	48 (27.3)	44 (23.2)
>137 102	102 (24.7)	54 (25.6)	48 (23.8)	47 (26.7)	46 (24.2)
Insurance status					
Commercial	246 (59.6)	136 (64.5)	110 (54.5)	118 (67.1)	106 (55.8)
Medicaid	72 (17.4)	31 (14.7)	41 (20.3)	19 (10.8)	39 (20.5)
Medicare	93 (22.5)	43 (20.4)	50 (24.8)	38 (21.6)	44 (23.2)
Self-pay or missing	2 (0.5)	1 (0.5)	1 (0.5)	1 (0.6)	1 (0.5)
Employment status					
Employed	235 (56.9)	132 (62.6)	103 (51.0)	112 (63.6)	100 (52.6)
Not employed	93 (22.5)	34 (16.1)	59 (29.2)	26 (14.8)	51 (26.8)
Retired	51 (12.3)	28 (13.3)	23 (11.4)	24 (13.6)	22 (11.6)
Student	14 (3.4)	7 (3.3)	7 (3.5)	5 (2.8)	7 (3.7)
Missing	20 (4.8)	10 (4.7)	10 (5.0)	9 (5.1)	10 (5.3)
Study clinic					
1	33 (8.0)	18 (8.5)	15 (7.4)	11 (6.3)	14 (7.4)
2	48 (11.6)	23 (10.9)	25 (12.4)	22 (12.5)	23 (12.1)
3	79 (19.1)	41 (19.4)	38 (18.8)	36 (20.5)	34 (17.9)
4	120 (29.1)	61 (28.9)	59 (29.2)	50 (28.4)	56 (29.5)
5	52 (12.6)	28 (13.3)	24 (11.9)	21 (11.9)	22 (11.6)
6	37 (9.0)	18 (8.5)	19 (9.4)	15 (8.5)	19 (10.0)
7	44 (10.7)	22 (10.4)	22 (10.9)	21 (11.9)	22 (11.6)
PCP type					
Physician	404 (97.8)	206 (97.6)	198 (99.0)	173 (98.3)	186 (98.9)
Nurse practitioner	7 (1.7)	5 (2.4)	2 (1.0)	3 (1.7)	2 (1.1)
Missing	2 (0.0)	NA	2 (1.0)	NA	2 (1.1)
Patient portal status					
Activated	393 (100)	202 (95.7)	191 (94.6)	171 (97.2)	180 (94.7)
Log-in date within 6 mo of study start	95 (23.6)	49 (23.2)	46 (22.8)	39 (22.2)	42 (22.1)
Smartphone type					
1 (Android)	101 (24.5)	47 (22.3)	54 (26.7)	40 (22.7)	51 (26.8)
2 (iPhone)	279 (67.6)	141 (66.8)	138 (68.3)	127 (72.2)	132 (69.5)
3 (Other)	4 (1.0)	4 (1.9)	0	4 (2.3)	0
Do not know or missing	29 (7.0)	19 (9.0)	10 (5.0)	5 (2.8)	7 (3.7)
Asthma severity					
Smoking status					
Current smoker	24 (5.8)	9 (4.3)	15 (7.4)	8 (4.6)	14 (7.4)
Former smoker	117 (28.4)	62 (29.5)	55 (27.2)	48 (27.4)	52 (27.4)
Never smoker	271 (65.8)	139 (66.2)	132 (65.4)	119 (68.0)	124 (65.3)
Missing	NA	1 (0.5)	NA	1 (0.6)	NA
Asthma exacerbation events (≥1) in prior 12 mo	130 (31.5)	68 (32.2)	62 (30.7)	59 (33.5)	59 (31.1)
Environmental, seasonal allergy, or allergic rhinitis comorbidity	396 (95.9)	204 (96.7)	192 (95.1)	171 (97.2)	180 (94.7)
General health					
CCI score, mean (SD)	1.7 (1.5)	1.7 (1.4)	1.8 (1.6)	1.7 (1.4)	1.8 (1.7)
BMI, mean (SD)	30.8 (7.8)	31.2 (8.0)	30.5 (7.7)	30.8 (8.0)	30.6 (7.8)
ACM, mean (SD)	5.0 (3.9)	4.9 (4.0)	5.1 (3.8)	4.6 (4.0)	4.9 (3.7)
PAM score level					
1	18 (4.4)	8 (3.8)	10 (5.0)	7 (4.0)	9 (4.7)
2	36 (8.8)	18 (8.6)	18 (8.9)	10 (5.7)	15 (7.9)
3	123 (29.9)	67 (32.1)	56 (27.7)	60 (34.1)	53 (27.9)
4	234 (57.0)	116 (55.5)	118 (58.4)	99 (56.3)	113 (59.5)
Missing	NA	2 (0.9)	NA	NA	NA
Health literacy (SLS score), mean (SD)	13.8 (2.2)	13.8 (2.0)	13.7 (2.4)	14.0 (1.8)	13.8 (2.4)
Questionnaire completion					
Time between baseline and 12-mo MiniAQLQ, mean (SD), d	NA	NA	NA	379.4 (36.9)	381.4 (34.8)
Time between baseline and 12-mo health care utilization, mean (SD), d	NA	NA	NA	380.4 (38.6)	380.1 (36.6)
MiniAQLQ score, mean (SD)^c^	5.11 (1.24)	5.18 (1.21)	5.04 (1.27)	5.34 (1.16)	5.08 (1.28)

Abbreviations: ACM, Asthma Control Measure; BMI, body mass index (calculated as weight in kilograms divided by height in meters squared); CCI, Charlson Comorbidity Index; GED, General Educational Development; MiniAQLQ, Mini Asthma Quality of Life Questionnaire; NA, not applicable; PAM, Patient Activation Measure; PCP, primary care practitioner; SLS, Short Health Literacy Survey.

^a^ Unless indicated otherwise, values are presented as No. (%) of patients.

^b^ Includes American Indian or Alaska Native, Asian, Native Hawaiian or Other Pacific Islander, or declined to answer.

^c^ MiniAQLQ scores range from 1 (worst) to 7 (best).

### Outcomes

#### Primary and Secondary Outcomes

In the primary analysis, mean MiniAQLQ scores increased more from baseline to 12 months in the intervention group (0.34 [95% CI, 0.19-0.49]) compared with the usual care group (0.11 [95% CI, −0.11 to 0.33]) (adjusted difference-in-difference, 0.23 [95% CI, 0.06-0.40]; *P* = .01) (Table 2). The difference was not clinically significant (defined as a 0.5-point increase). The sensitivity analysis with imputation of the missing final MiniAQLQ questionnaire response (eTable 3 in Supplement 2) found a slightly greater and still significant difference in change in MiniAQLQ score from baseline to 12 months between the intervention group (0.37 [95% CI, 0.25-0.49]) compared with the usual care group (0.11 [95% CI, −0.01 to 0.23]) (adjusted difference-in-difference, 0.26 [95% CI, 0.09-0.43]; *P* < .01).

**Table 2.** Effect of Intervention on Asthma-Related Quality of Life (Primary Analysis)^a^

MiniAQLQ score^b^	Intervention group (n = 176)	Usual care group (n = 190)	Difference between groups	*P* value
Baseline, mean (95% CI)	5.31 (5.14-5.48)	5.25 (5.10-5.40)	NA	NA
At 12 mo, mean (95% CI)	5.65 (5.51-5.79)	5.36 (5.21-5.50)	NA	NA
Change from baseline, effect size (95% CI)	0.34 (0.19-0.49)	0.11 (−0.11 to 0.33)	0.23 (0.06-0.40)	.01

Abbreviations: MiniAQLQ, Mini Asthma Quality of Life Questionnaire; NA, not applicable.

^a^ Values are clustered by primary care practitioner and adjusted for sex, income, insurance, employment, education, and timing of completion of the 12-month MiniAQLQ.

^b^ MiniAQLQ scores range from 1 (worst) to 7 (best).

In the secondary analysis, nonroutine asthma-related utilization events were measured as the total number of asthma-related emergency department visits, urgent care visits, and hospitalizations (with asthma in the primary or secondary position) at MGB–affiliated institutions identified in the EHR during the study period per patient. After clustering by primary care practitioner and adjusting for sex, income, insurance, employment, and education, the mean number of events did not differ between the 211 patients in the intervention group (0.59 [95% CI, 0.42-0.77]) and the 202 patients in the usual care group (0.76 [95% CI, 0.55-0.96]) (adjusted effect size, −0.16 [95% CI, −0.42 to 0.17]; *P* = .23). Sensitivity analyses with patients who completed only the 12-month MiniAQLQ and including only patient-reported data on asthma-related health care utilization showed similar results (eTables 4 and 5 in Supplement 2).

#### Subgroup and Exploratory Analyses

**Figure 2.**
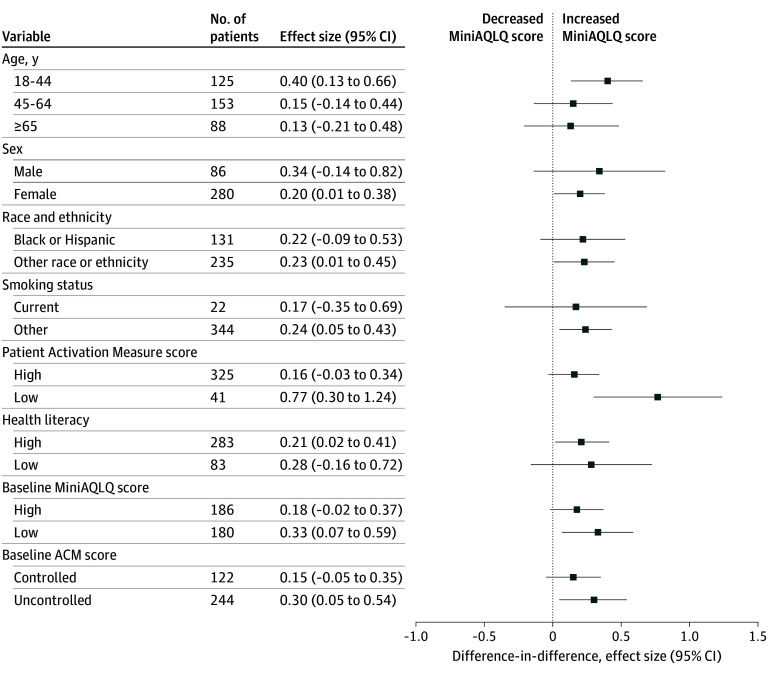
Subgroup Analysis of Intervention Effect on Asthma-Related Quality of Life The difference-in-difference in Mini Asthma Quality of Life Questionnaire (MiniAQLQ) scores is presented. ACM indicates Asthma Control Measure.

All subgroups showed increases in MiniAQLQ scores (Figure 2 and eTable 6 in Supplement 2). Subgroups showing a noteworthy increase included patients aged 18 to 44 years (adjusted difference-in-difference, 0.40 [95% CI, 0.13-0.66]); those with low baseline PAM scores, defined as levels 1 or 2 (adjusted difference-in-difference, 0.77 [95% CI, 0.30-1.24]); those with low baseline MiniAQLQ scores, defined as below the median (adjusted difference-in-difference, 0.33 [95% CI, 0.07-0.59]); and those with uncontrolled asthma at baseline, as indicated by ACM scores (adjusted difference-in-difference, 0.30 [95% CI, 0.05-0.54]).

In exploratory analyses, the mean change in MiniAQLQ subdomain score showed greater increases in the intervention group that were noteworthy for 3 of the MiniAQLQ subdomains (eTable 7 in Supplement 2). These subdomains included exposure to environmental stimuli (adjusted difference-in-difference, 0.35 [95% CI, 0.10-0.59]), emotional function (adjusted difference-in-difference, 0.30 [95% CI, 0.03-0.57]), and symptoms (adjusted difference-in-difference, 0.25 [95% CI, 0.04-0.46]). The mean change in subdomain score for the activity limitations subdomain was less noteworthy (adjusted difference-in-difference, 0.10 [95% CI, −0.11 to 0.30]).

There were no noteworthy differences between the intervention and usual care groups in mean number of asthma-related outpatient visits (adjusted effect size, 0.35 [95% CI, −0.10 to 0.80]) (eTable 8 in Supplement 2) or prednisone prescriptions (odds ratio, 1.03 [95% CI, 0.62-1.69]) (eTable 9 in Supplement 2). Repeated-measures analysis showed that a larger increase in MiniAQLQ score due to the intervention occurred in the first 6 months of the study compared with the second 6 months (eTable 10 in Supplement 2). Analysis of patients with varying levels of intervention adherence and retention as defined by app questionnaire completion rates (eTable 11 in Supplement 2) showed that patients with low adherence had a greater increase in mean MiniAQLQ score (0.68 [95% CI, 0.36-1.00]) compared with those with high adherence (0.25 [95% CI, 0.13-0.36]), and patients with low retention had a greater increase in mean MiniAQLQ score (0.51 [95% CI, 0.23-0.79]) compared with those with high retention (0.26 [95% CI, 0.14-0.39]).

## Discussion

In this study, patients using a scalable between-visit asthma symptom monitoring intervention had a greater but nonclinically significant increase in patient-reported asthma-related quality of life over the study period compared with patients receiving usual care. The sensitivity analysis using multiple imputation for missing data showed similar results. Clinically meaningful increases in asthma-related quality of life were found in subgroup analyses for patients with lower baseline levels of activation, as measured by PAM scores and post hoc analyses of patients with lower levels of adherence and retention in questionnaire completion. In terms of MiniAQLQ subdomains assessing asthma-related quality of life, scores for the exposure to environmental stimuli subdomain showed the largest increase in the intervention group compared with the usual care group, followed by scores for the emotional function and symptom subdomains; scores for the activity limitations subdomain did not show a noteworthy difference. We did not find differences in secondary analyses or sensitivity analyses looking at asthma-related health care utilization. Overall, these data suggest that between-visit symptom monitoring led to an average improvement in asthma quality of life among study patients, although this improvement did not meet the threshold of a minimally important change. However, the intervention showed potential benefits for certain subgroups and may be useful in other chronic diseases in which between-visit symptoms are important, such as rheumatoid disorders, musculoskeletal disorders, and mental health conditions.^35,36,37,38,39,40,41,42,43^

The primary outcome showing an increase in asthma-related quality of life is consistent with prior studies that evaluated asthma symptom monitoring interventions. Specifically, 2 studies in the Netherlands found statistically significant effect-size increases in asthma-related quality of life (0.38^12,44^ and 0.29^13^) for asthma symptom monitoring compared with our study, which found an effect size of 0.23 (95% CI, 0.06-0.40) on comparable scales. Of note, the interventions in those studies included less scalable components (eg, nurse-initiated web communication with an asthma nurse, a personal asthma action plan, and group education sessions). Other studies of symptom monitoring are less scalable because they depend on devices that patients do not already own, such as digital inhalers.^15,45,46,47^ It is likely that we achieved comparable results with a more scalable intervention, in part, due to our user-centered design process^18^ and use of the NASSS implementation framework.^21,22^ Our finding that the intervention has the largest effect in the first few months is consistent with patterns reported in other studies that monitor asthma symptoms over time.^11^ The increase in MiniAQLQ scores for patients with low patient activation compared with usual care is a novel contribution and suggests that additional outreach to this subgroup of patients is warranted.

In our previous user-centered design work and pilot testing of the intervention used in this study, we found that the intervention increased patient awareness of their asthma symptoms, increased their feeling of connection with their care teams, and facilitated increased clinical support, allowing patients to address issues more quickly.^19,20^ It is likely that the greater, although not clinically significant, increase observed in asthma-related quality of life among intervention patients reflects improvements in asthma self-management and interactions with care teams when needed among this group of patients. The relatively larger effects on patients with low activation (and low adherence, which may be related to activation) may be because those patients have more potential to improve their knowledge, skills, and confidence in managing their health, and this intervention helps them with those factors.

### Limitations

Our study has several limitations. First, study patients at baseline had higher scores on our primary outcome compared with those of other studies in which patient improvement was clinically significant.^48^ Had we recruited patients with worse baseline scores, the differential improvement may have reached clinical significance. Second, data were missing for 47 participants (11.4%) in this study, with some differences in characteristics of those lost to follow-up between groups, potentially introducing bias. We addressed this issue by adjusting for differences in baseline covariates and conducting a sensitivity analysis with multiple imputation. Third, because masking study group assignment was not feasible, there is a risk of patients overreporting or underreporting symptoms. Fourth, as prior studies suggest,^11^ simply enrolling patients in a study may improve asthma outcome measures in all study groups because patients are more aware of their asthma. We tried to minimize this effect by only sending patient questionnaires at baseline, 6 months, and 12 months (as opposed to monthly, which is common in asthma studies), but those interactions may have made patients more aware of their asthma in the usual care group, which would bias the study toward a null finding. Fifth, the study was implemented during the COVID-19 pandemic, which may differ from nonpandemic times and added barriers to recruitment. Specifically, there have been decreases in asthma exacerbations,^49^ asthma-related emergency department utilization,^50^ and overall emergency department utilization attributed to the pandemic and pandemic-related public health measures (eg, lockdowns) that may have also resulted in greater scores in our primary outcome measures among all patients, attenuating differences across groups. It is notable that even with smaller numbers of patients than our target, the primary outcome was still statistically significant. We may have been underpowered for our secondary outcomes because we did not achieve our target recruitment goals. Finally, the intervention was implemented in the primary care setting; specialists treating enrolled patients were not engaged.

## Conclusions

In this RCT of a scalable asthma symptom monitoring intervention, increases in asthma-related quality of life did not reach the threshold of a minimally important change, and no changes were observed in nonroutine asthma-related health care utilization. However, subgroups of patients, particularly those with lower levels of activation, may derive meaningful benefits and should be the focus of further development and testing.

## Notes

Supplement 1.
Trial Protocol and Statistical Analysis Plan

Supplement 2. **eAppendix 1.** Intervention Components **eAppendix 2.** Survey Instruments Administered **eTable 1.** Baseline Patient Characteristics Stratified by Final Survey Completion Status **eTable 2.** Patients per Primary Care Physician Stratified by Patient Final Survey Completion Status **eTable 3.** Primary Analysis With Imputation of Missing 12-Month MiniAQLQ (Sensitivity Analysis) **eTable 4.** Secondary Analysis With Only Patients Who Completed 12-Month MiniAQLQ (Sensitivity Analysis) **eTable 5.** Secondary Outcome Analysis of Patient-Reported Utilization Internal and External to Health System (Sensitivity Analysis) **eTable 6.** Subgroup Analysis **eTable 7.** Patient Responses to Subdomains of the MiniAQLQ by Treatment Group (Exploratory) **eTable 8.** Outpatient Utilization by Treatment Group (Exploratory) **eTable 9.** Prednisone Prescriptions by Treatment Group (Exploratory) **eTable 10.** Repeat Measures Analyses (Exploratory) **eTable 11.** Adherence and Retention for Intervention Group Patients

Supplement 3.
Data Sharing Statement

## References

[1] GBD cause and risk summaries. Global Burden of Disease. *The Lancet*. Accessed October 24, 2023. https://www.thelancet.com/gbd/summaries

[2] Most recent national asthma data. US Centers for Disease Control and Prevention. Accessed October 24, 2023. https://www.cdc.gov/asthma/most_recent_national_asthma_data.htm

[3] National Surveillance of Asthma. United States, 2001-2010. November 2012. National Center for Health Statistics. Accessed March 14, 2025. https://www.cdc.gov/nchs/data/series/sr_03/sr03_035.pdf 24252609

[4] Szentpetery SE, Forno E, Canino G, Celedón JC. Asthma in Puerto Ricans: lessons from a high-risk population. J Allergy Clin Immunol. 2016;138(6):1556-1558. doi:10.1016/j.jaci.2016.08.047 27751794 PMC5189666

[5] Bartolomei-Díaz JA, Amill-Rosario A, Claudio L, Hernández W. Asthma mortality in Puerto Rico: 1980-2007. J Asthma. 2011;48(2):202-209. doi:10.3109/02770903.2010.528498 21039188 PMC3190980

[6] National Asthma Education and Prevention Program, Third Expert Panel on the Diagnosis and Management of Asthma. Expert Panel Report 3: Guidelines for the Diagnosis and Management of Asthma. National Heart, Lung, and Blood Institute. 2007. Accessed April 11, 2021. https://www.ncbi.nlm.nih.gov/books/NBK7232/

[7] Lang DM. New asthma guidelines emphasize control, regular monitoring. Cleve Clin J Med. 2008;75(9):641-653. doi:10.3949/ccjm.75.9.641 18788225

[8] Cloutier MM, Baptist AP, Blake KV, ; Expert Panel Working Group of the National Heart, Lung, and Blood Institute (NHLBI) administered and coordinated National Asthma Education and Prevention Program Coordinating Committee (NAEPPCC). 2020 Focused updates to the asthma management guidelines: a report from the National Asthma Education and Prevention Program Coordinating Committee Expert Panel Working Group. J Allergy Clin Immunol. 2020;146(6):1217-1270. doi:10.1016/j.jaci.2020.10.003 33280709 PMC7924476

[9] Hui CY, Walton R, McKinstry B, Jackson T, Parker R, Pinnock H. The use of mobile applications to support self-management for people with asthma: a systematic review of controlled studies to identify features associated with clinical effectiveness and adherence. J Am Med Inform Assoc. 2017;24(3):619-632. doi:10.1093/jamia/ocw143 27694279 PMC7651908

[10] Yildiz F; ASIT Study Group. Factors influencing asthma control: results of a real-life prospective observational Asthma Inhaler Treatment (ASIT) study. J Asthma Allergy. 2013;6:93-101. doi:10.2147/JAA.S45269 23843695 PMC3702275

[11] Israel E, Cardet JC, Carroll JK, . Reliever-triggered inhaled glucocorticoid in Black and Latinx adults with asthma. N Engl J Med. 2022;386(16):1505-1518. doi:10.1056/NEJMoa2118813 35213105 PMC10367430

[12] van der Meer V, Bakker MJ, van den Hout WB, ; SMASHING (Self-Management in Asthma Supported by Hospitals, ICT, Nurses and General Practitioners) Study Group. Internet-based self-management plus education compared with usual care in asthma: a randomized trial. Ann Intern Med. 2009;151(2):110-120. doi:10.7326/0003-4819-151-2-200907210-00008 19620163

[13] van Gaalen JL, Beerthuizen T, van der Meer V, ; SMASHING Study Group. Long-term outcomes of internet-based self-management support in adults with asthma: randomized controlled trial. J Med Internet Res. 2013;15(9):e188. doi:10.2196/jmir.2640 24028826 PMC3785973

[14] Mosnaim G, Safioti G, Brown R, . Digital health technology in asthma: a comprehensive scoping review. J Allergy Clin Immunol Pract. 2021;9(6):2377-2398. doi:10.1016/j.jaip.2021.02.028 33652136

[15] Merchant RK, Inamdar R, Quade RC. Effectiveness of population health management using the Propeller Health asthma platform: a randomized clinical trial. J Allergy Clin Immunol Pract. 2016;4(3):455-463. doi:10.1016/j.jaip.2015.11.022 26778246

[16] Araújo L, Jacinto T, Moreira A, . Clinical efficacy of web-based versus standard asthma self-management. J Investig Allergol Clin Immunol. 2012;22(1):28-34. 22448451

[17] Demographics of mobile device ownership and adoption in the United States. Pew Research Center. Accessed October 24, 2023. https://www.pewresearch.org/internet/fact-sheet/mobile/

[18] Rudin RS, Perez S, Rodriguez JA, . User-centered design of a scalable, electronic health record-integrated remote symptom monitoring intervention for patients with asthma and providers in primary care. J Am Med Inform Assoc. 2021;28(11):2433-2444. doi:10.1093/jamia/ocab157 34406413 PMC8510383

[19] Rudin RS, Fanta CH, Qureshi N, . A clinically integrated mHealth app and practice model for collecting patient-reported outcomes between visits for asthma patients: implementation and feasibility. Appl Clin Inform. 2019;10(5):783-793. doi:10.1055/s-0039-1697597 31618782 PMC6795530

[20] Rudin RS, Fanta CH, Predmore Z, . Core components for a clinically integrated mHealth app for asthma symptom monitoring. Appl Clin Inform. 2017;8(4):1031-1043. doi:10.4338/ACI-2017-06-RA-0096 29241243 PMC5802299

[21] Greenhalgh T, Wherton J, Papoutsi C, . Analysing the role of complexity in explaining the fortunes of technology programmes: empirical application of the NASSS framework. BMC Med. 2018;16(1):66. doi:10.1186/s12916-018-1050-6 29754584 PMC5950199

[22] Greenhalgh T, Wherton J, Papoutsi C, . Beyond adoption: a new framework for theorizing and evaluating nonadoption, abandonment, and challenges to the scale-up, spread, and sustainability of health and care technologies. J Med Internet Res. 2017;19(11):e367. doi:10.2196/jmir.8775 29092808 PMC5688245

[23] Sulca Flores JA, Dalal AK, Sousa J, . Evaluation of a primary care-integrated mobile health intervention to monitor between-visit asthma symptoms. Appl Clin Inform. 2024;15(4):785-797. doi:10.1055/s-0044-1788978 39357877 PMC11446627

[24] Amagai S, Pila S, Kaat AJ, Nowinski CJ, Gershon RC. Challenges in participant engagement and retention using mobile health apps: literature review. J Med Internet Res. 2022;24(4):e35120. doi:10.2196/35120 35471414 PMC9092233

[25] Harris PA, Taylor R, Minor BL, ; REDCap Consortium. The REDCap consortium: building an international community of software platform partners. J Biomed Inform. 2019;95:103208. doi:10.1016/j.jbi.2019.103208 31078660 PMC7254481

[26] Hibbard JH, Stockard J, Mahoney ER, Tusler M. Development of the Patient Activation Measure (PAM): conceptualizing and measuring activation in patients and consumers. Health Serv Res. 2004;39(4 pt 1):1005-1026. doi:10.1111/j.1475-6773.2004.00269.x 15230939 PMC1361049

[27] Hibbard JH, Mahoney ER, Stockard J, Tusler M. Development and testing of a short form of the patient activation measure. Health Serv Res. 2005;40(6 pt 1):1918-1930. doi:10.1111/j.1475-6773.2005.00438.x 16336556 PMC1361231

[28] Kearns R, Harris-Roxas B, McDonald J, Song HJ, Dennis S, Harris M. Implementing the Patient Activation Measure (PAM) in clinical settings for patients with chronic conditions: a scoping review. Integr Healthc J. 2020;2(1):e000032. 37441314 10.1136/ihj-2019-000032 PMC10327461

[29] Juniper EF, Guyatt GH, Cox FM, Ferrie PJ, King DR. Development and validation of the Mini Asthma Quality of Life Questionnaire. Eur Respir J. 1999;14(1):32-38. doi:10.1034/j.1399-3003.1999.14a08.x 10489826

[30] Charlson ME, Carrozzino D, Guidi J, Patierno C. Charlson Comorbidity Index: a critical review of clinimetric properties. Psychother Psychosom. 2022;91(1):8-35. doi:10.1159/000521288 34991091

[31] Jakobsen JC, Gluud C, Wetterslev J, Winkel P. When and how should multiple imputation be used for handling missing data in randomised clinical trials—a practical guide with flowcharts. BMC Med Res Methodol. 2017;17(1):162. doi:10.1186/s12874-017-0442-1 29207961 PMC5717805

[32] Groenwold RHH, Moons KGM, Vandenbroucke JP. Randomized trials with missing outcome data: how to analyze and what to report. CMAJ. 2014;186(15):1153-1157. doi:10.1503/cmaj.131353 24778353 PMC4203602

[33] Fielding S, Maclennan G, Cook JA, Ramsay CR. A review of RCTs in four medical journals to assess the use of imputation to overcome missing data in quality of life outcomes. Trials. 2008;9(1):51. doi:10.1186/1745-6215-9-51 18694492 PMC3225816

[34] White IR, Horton NJ, Carpenter J, Pocock SJ. Strategy for intention to treat analysis in randomised trials with missing outcome data. BMJ. 2011;342(7803):d40. doi:10.1136/bmj.d40 21300711 PMC3230114

[35] Seppen BF, den Boer P, Wiegel J, . Asynchronous mHealth interventions in rheumatoid arthritis: systematic scoping review. JMIR Mhealth Uhealth. 2020;8(11):e19260. doi:10.2196/19260 33151161 PMC7677027

[36] Lee YC, Lu F, Colls J, . Outcomes of a mobile app to monitor patient-reported outcomes in rheumatoid arthritis: a randomized controlled trial. Arthritis Rheumatol. 2021;73(8):1421-1429. doi:10.1002/art.41686 33559338 PMC8330418

[37] Solomon DH, Dalal AK, Landman AB, . Development and testing of an electronic health record-integrated patient-reported outcome application and intervention to improve efficiency of rheumatoid arthritis care. ACR Open Rheumatol. 2022;4(11):964-973. doi:10.1002/acr2.11498 36099161 PMC9661861

[38] Solomon DH, Altwies H, Santacroce L, . A mobile health application integrated in the electronic health record for rheumatoid arthritis patient-reported outcomes: a controlled interrupted time-series analysis of impact on visit efficiency. Arthritis Rheumatol. 2024;76(5):677-683. doi:10.1002/art.42774 38087859

[39] Solomon DH, Rudin RS. Digital health technologies: opportunities and challenges in rheumatology. Nat Rev Rheumatol. 2020;16(9):525-535. doi:10.1038/s41584-020-0461-x 32709998

[40] Jiang Y, Rudin RS, Solomon DH. Clinical visit frequencies in rheumatology: a systematic literature review. Arthritis Care Res (Hoboken). 2023;75(10):2054-2062. doi:10.1002/acr.25106 36807719 PMC10440366

[41] Rudin RS, Garabedian PM, Solomon DH. Know thy user: designing digital tools for rheumatology patients. J Rheumatol. 2024;51(9):845-847. doi:10.3899/jrheum.2024-0684 39089841

[42] Rudin RS, Friedberg MW, Solomon DH. In the COVID-19 era and beyond, symptom monitoring should be a universal health care function. *Health Affairs Forefront*. June 18, 2020. Accessed March 14, 2025. https://www.healthaffairs.org/content/forefront/covid-19-era-and-beyond-symptom-monitoring-should-universal-health-care-function

[43] Colls J, Lee YC, Xu C, . Patient adherence with a smartphone app for patient-reported outcomes in rheumatoid arthritis. Rheumatology (Oxford). 2021;60(1):108-112. doi:10.1093/rheumatology/keaa202 32572490

[44] van der Meer V, van Stel HF, Bakker MJ, ; SMASHING (Self-Management of Asthma Supported by Hospitals, ICT, Nurses and General practitioners) Study Group. Weekly self-monitoring and treatment adjustment benefit patients with partly controlled and uncontrolled asthma: an analysis of the SMASHING study. Respir Res. 2010;11(1):74. doi:10.1186/1465-9921-11-74 20537124 PMC2893513

[45] Buist AS, Vollmer WM, Wilson SR, Frazier EA, Hayward AD. A randomized clinical trial of peak flow versus symptom monitoring in older adults with asthma. Am J Respir Crit Care Med. 2006;174(10):1077-1087. doi:10.1164/rccm.200510-1606OC 16931634 PMC2648108

[46] Merchant R, Szefler SJ, Bender BG, . Impact of a digital health intervention on asthma resource utilization. World Allergy Organ J. 2018;11(1):28. doi:10.1186/s40413-018-0209-0 30524644 PMC6276132

[47] Silberman J, Sarlati S, Harris B, . A digital approach to asthma self-management in adults: protocol for a pragmatic randomized controlled trial. Contemp Clin Trials. 2022;122:106902. doi:10.1016/j.cct.2022.106902 36049674 PMC12582168

[48] Licskai C, Sands TW, Ferrone M. Development and pilot testing of a mobile health solution for asthma self-management: asthma action plan smartphone application pilot study. Can Respir J. 2013;20(4):301-306. doi:10.1155/2013/906710 23936890 PMC3956342

[49] Shah SA, Quint JK, Sheikh A. Impact of COVID-19 pandemic on asthma exacerbations: retrospective cohort study of over 500,000 patients in a national English primary care database. Lancet Reg Health Eur. 2022;19:100428. doi:10.1016/j.lanepe.2022.100428 35756853 PMC9213032

[50] Boserup B, McKenney M, Elkbuli A. The impact of the COVID-19 pandemic on emergency department visits and patient safety in the United States. Am J Emerg Med. 2020;38(9):1732-1736. doi:10.1016/j.ajem.2020.06.007 32738468 PMC7274994

