# Supplementary material for: Between-Visit Asthma Symptom Monitoring With a Scalable Digital Intervention: A Randomized Clinical Trial
Source: JAMA Netw Open. 2025 Apr 23;8(4):e256219. doi: 10.1001/jamanetworkopen.2025.6219 (PMC12019512; doi:10.1001/jamanetworkopen.2025.6219)
Supplement: Supplement 1. — Trial Protocol and Statistical Analysis Plan [file jamanetwopen-e256219-s001.pdf]

Supplement 1: Trial protocol and statistical analysis plan

PROTOCOL TITLE

Integrating Patient-Reported Outcomes into Routine Primary Care: Monitoring Asthma Between Visits

FUNDING

AHRQ (1R18HS026432-01)

VERSION DATE

03/03/23

I. BACKGROUND AND SIGNIFICANCE

Asthma affects more than 25 million individuals in the United States, and its incidence is increasing.[1] Uncontrolled asthma causes substantial suffering (disproportionately among minorities and those of lower socioeconomic status),[2] and often results in the use of emergency medical services and/or hospitalization.[3] Poor asthma control may significantly degrade quality of daily life, impairing daily physical activities, sleep, and ability to work. Yet poor health outcomes from inadequate asthma control are preventable, especially with the provision of more timely treatment and adherence to clinical practice guidelines that call for clinicians to adjust treatment based on serial monitoring of patients' reported symptoms.[1 4-8] In fact, adhering to clinical guidelines has been shown to improve health outcomes that are important to patients.[7, 9] In contrast to relying on clinical measures such as lung function (which do not directly reflect patients' lived experience) routine collection of asthma symptoms in the form of patient-reported outcomes (PROs) could focus treatment on what matters most to patients–their symptoms and quality of life. Unfortunately, intensive symptom monitoring and serial measurement is largely not happening.[10, 11] Though clinicians are increasingly collecting asthma-related PROs immediately prior to a patient visit, there has been little progress in collecting PROs during the interval *between* visits. Interventions that facilitate monitoring of asthma symptoms during this interval offer a promising strategy to improve symptom control and quality of life, as well as reduce healthcare utilization for patients with asthma.

In our previous AHRQ-funded pilot, we demonstrated the success of a simple health IT-enabled practice model developed to facilitate asthma symptom monitoring via a clinically integrated mHealth app installed on patients' smartphones during the interval between subspecialty clinic visits in the ambulatory setting. We used an iterative, user-centered design process to ensure our health IT tools maximally engage patients between visits, and to identify optimal clinic practice workflow to minimize burden on clinicians.[12] Preliminary data from our 6-month pilot suggest strong engagement: of 26 patients of pulmonologists, 24 (92%) continued to use the app after 4-6 months, and 18 (69%) had 100% adherence to completion of the weekly questionnaires. Exit

interviews show positive support, and patients feel that the intervention facilitates symptom tracking, self-awareness of symptoms, and more timely access to a clinician when symptoms worsen. As smartphone adoption—now exceeding 81% of the U.S. population—continues to increase, an mHealth strategy for collecting PROs as we describe is ready for scale and spread. Specifically, an intervention comprised of our health IT-enabled practice model is well-suited to be adapted to the primary care setting to facilitate PRO collection between visits for a larger population of patients with the goal of improving guideline-concordant monitoring of asthma symptoms.

We are adapting our novel practice model to the primary care setting within an accountable care organization that includes a large and diverse population of asthma patients, including Spanish-speaking and low health literacy patients. Specifically, we will work with 4 primary care clinics affiliated with Brigham Health's Practice-Based Research Network. To adapt our practice model, we will use a novel framework designed specifically to scale and spread healthcare technology innovations—Non-adoption, Abandonment, Scale-up, Spread, and Sustainability (NASSS). This framework emphasizes the importance of simplicity in designing technology and practice models to scalability. We will work closely with the primary care clinics to understand their requirements, adapt our technology, and simplify workflows as per each dimension of the NASSS framework. We will then implement our adapted practice model at participating clinics and conduct a rigorous evaluation to understand the impact on key asthma-related health outcomes.

If successful, this project will provide new knowledge about how to scale and spread a health IT-enabled practice model that incorporates the collection asthma-related PROs into routine primary care, the setting in which most asthma patients are treated. This work is innovative because it adapts an existing and proven health IT-enabled practice model that leverages widespread adoption of smartphones among patients and is guided by a novel framework specifically designed to scale and spread health technology innovations. Furthermore, our findings will produce rigorous evidence regarding impact, thereby providing a foundation for developing a set of best practices to scale and spread this or similar health IT-enabled practice models for between-visit collection of PROs for patients with asthma as well as other conditions.

II. SPECIFIC AIMS

1. Adapt our existing health IT-enabled practice model for asthma symptom monitoring using patient-reported outcome (PROs) to a primary care population.

2. Implement the adapted health IT-enabled practice model in 7 primary care community clinics, identify a cohort of eligible asthma patients to participate, and train clinicians and clinical staff.

3. Rigorously evaluate the impact of this new health IT-enabled practice model using a randomized controlled trial study in which we enroll up to 750 asthma patients (375 intervention, 375 usual care) by primary care clinician.

III. SUBJECT SELECTION

<u>Phase I – Design Sessions</u>

During Phase I, any male or female, English-speaking patient (>18 years of age) able to provide verbal consent will be eligible. Any male or female healthcare provider (physicians and nurses) who is at least 18 years of age is eligible to participate. Additionally, we will recruit a cohort of people during Phase I to participate in further validation of the Asthma Control Measure (ACM).

We will use four different strategies to recruit patients for design sessions during Phase I:

1. PCPs Primary care physicians will give their approval to contact patients on their respective panels;
2. Medical directors will give their approval to contact patients in their respective clinics;
3. Care team members will give approval to recruit patients from Medicine floors who are nearing discharge to home; and
4. IRB-approved flyer or Rally advertisement.

We will work with clinic leads to identify physicians. Research assistants, trained in the protection of human subjects, will identify eligible patients by looking through participating physicians' panels for patients who have asthma and using a purposeful sample. The study team will then ask for permission from the physician or the clinic medical director before contacting the patient. Participating BWH/BWFH physicians may also recommend patients from their respective panels who may be eligible. The PI (Dr. Dalal) will then mail the patients two letters (one signed by the patient's primary care physician or clinic medical director and one signed by the PI) inviting them to join the study.

We will also approach hospitalized patients admitted with asthma or asthma-comparable illness (e.g., COPD) from Medicine floors who are nearing discharge to home. Patients will be identified from administrative databases and recruited in-person after getting approval from the patient's nurse or member of the care team

ACM validation survey participants will be recruited through the Rally website.

<u>Phase II - RCT</u>

During Phase II, we will recruit participants according to the inclusion and exclusion criteria specified below.

Inclusion Criteria

- ☐ English- or Spanish-speaking
- ☐ >18-years of age
- ☐ Regularly uses (most days) a compatible smartphone (iOS or Android)
- ☐ Able to provide consent
- ☐ Seen for primary care within the last 12 months at:
  - REDACTED
- ☐ At least one visit coded for asthma within 12 months prior to screening

Exclusion Criteria

- ☐ Deemed inappropriate for study, per judgment of BWH/BWFH primary care provider
- ☐ Unable to provide consent

We will use any of the following strategies to recruit patients during Phase II:

1. We will directly contact eligible patients who have opted-in via the Partners RODY (Research Opportunities Direct to You) program
2. We will obtain approval from Brigham Health's Primary Care Practice-Based Research Network (PBRN) Director to contact patients affiliated with each participating primary care clinic. Medical directors of each clinic have agreed to this process and primary care physicians will be provided a 7-day period to opt-out of having their patients contacted.
3. Care team members will give approval to recruit patients from Medicine floors who are nearing discharge to home.
4. IRB-approved flyer or Rally advertisement.

The research team will advertise the study using approved flyers and web advertisements on the Rally website to invite interested patients to contact us. We will also identify eligible asthma patients of participating clinics through review of administrative databases. PCPs may also elect to identify patients from their respective panels who have a diagnosis of asthma and meet our inclusion criteria. The PI (Dr. Dalal) will send the MyChart Recruitment Message to eligible patients through Patient Gateway, a PeC approved recruitment process. Additionally, eligible patients may be sent recruitment letters (from the PI and the PBRN Director) via mail inviting them to participate. We may also approach patients in-person in outpatient settings, after getting approval from the patient's PCP or the Clinic Medical Director or PBRN Director. Finally, we may approach hospitalized patients with a diagnosis of asthma who are nearing discharge to home. Patients will be identified from the EHR and/or administrative databases and recruited in-person after getting approval from the patient's nurse or member of the care team.

IV. SUBJECT ENROLLMENT

<u>Phase I – Design Sessions</u>

A research assistant will follow up with a phone call to patients. If the patient agrees to participate, the research assistant will schedule the interview. For hospitalized patients admitted with asthma or asthma-comparable illness (e.g., COPD) from Medicine floors who are nearing discharge to home, the nurse or care team member will introduce the study staff to the patient and gain permission of the patient to be approached by study staff. If the patient agrees to participate, we will verbally consent them for participation.

Prior to each interview, all physicians, nurses, and patient subjects will be read consent language and asked to provide verbal consent as well as permission to have the interview audio recorded. **Thus, for phase I patient subjects will be recruited if their physicians or clinic medical directors provide permission, are informed, and agree to participate in this study.**

Patient demographic data will be collected including age, gender, and race/ethnicity. Patients will be informed that they may decline or opt-out from participating at any time (see below).

Clinician demographic data will be collected including age, gender, race/ethnicity, years of experience, types of position in health care, years working in facility, education, etc. Clinicians will be informed that they may decline or opt-out from participating at any time.\

ACM validation survey participants will be recruited through Rally to participate in a two short surveys by email (via REDCap). The surveys contain a total of 10 questions and should take less than five minutes to complete. ACM validation survey participants will be provided with a consent statement to read at the start of the REDCap survey.

<u>Phase II - RCT</u>

A research assistant will follow-up with eligible patients by phone if necessary. In some cases, we may approach hospitalized patients with a diagnosis of asthma who are nearing discharge to home. Patients will be identified from the EHR and/or administrative databases and recruited in-person after getting approval from the patient's nurse or member of the care team. The nurse or care team member will introduce the study staff to the patient and gain permission of the patient to be approached by study staff.

Prior to randomization and enrollment in the study, patients will read the consent form in REDcap and provide oral consent via phone to participate.

All potentially eligible participants will complete a pre-screening questionnaire over the phone, in-person, or through REDCap. Potential participants that pass the screening questions will be asked to answer additional questions through REDcap, over the phone, or in-person about their asthma (e.g., asthma-related quality of life, patient activation, and health literacy) as well as demographics and healthcare utilization to inform eligibility for the study. Eligible patients who meet the study criteria will then contacted and consented by phone or in-person, and then randomized to the intervention or control group.

For patients randomized to the intervention group, a member of the study team will provide assistance with installing and using the mHealth app as required. Intervention patients will be prompted via the app on a weekly basis to complete a 5-item questionnaire about their asthma symptoms (the Asthma Control Measure). If the patients' symptom score meets specific criteria for severity, the app will allow the patient to request a call from a nurse in the clinic. The app will also allow the patient to enter notes, peak flows, and to review previous symptoms. The app will also provide other relevant health information such as educational videos. The patient-reported data will not be part of the legal electronic health record (EHR) but will be accessible from the EHR. The app will be approved by RISO. Patients randomized to usual care will not have access to the mHealth app.

V. STUDY PROCEDURES

<u>Phase I – Design Sessions</u>

In-person interviews or phone will be conducted with participants up to two times with an approved member of the research team. Participants will be asked about their experience with asthma, how a smartphone app might be helpful to them, and about our materials and workflows for the RCT. Some patients may be asked to complete the ACM, the Mini Asthma Quality of

Life Questionnaire (Mini AQLQ), Short Literacy Survey, Patient Activation Measure, RCT screening, and other surveys by email (via REDCap). Each meeting will last approximately one hour.

Additionally, we will administer the ACM and Asthma Control Questionnaire by email (via REDCap) with up to 400 people.

Phase II - RCT

Training. At the beginning of the intervention, patients will be trained to use the app via a brief in-app video that explains the key features. We will train the nurses in charge of responding to the notifications to use the aggregate dashboard to view their panel of enrolled patients (Appendix A). They will follow a triage script developed by our clinical team for our previous pilot. Finally, we will train the participating physicians to access the PRO data in the EHR from the patient's chart (Figure 2). We expect this training to be minimal.

Usual Care. Patients randomized to usual care will continue to receive care as prescribed by their PCP; they will not have access to the mHealth app.

Randomization. Eligible patients will be randomly allocated to the intervention or usual care. The random assignment will be made by computer-generated randomization to each treatment group by permuted blocks of a size of 2 within each clinician, ensuring that for all consecutive blocks of a size of 2 within each clinician, 1 subject will be randomized to the intervention and 1 to usual care.

Participants assigned to the control arm will be mailed asthma educational materials currently used at BWH. Participants assigned to the intervention arm will download the ASTHMA app to their smartphone. This app will send them notifications every week to prompt them to answer 5 standardized questions about their asthma symptoms. If their responses indicate severe or worsening symptoms, patients will be offered the option to request a call from a nurse within their clinic. The app will also contain a graph of participants' previous questionnaire responses, links to asthma educational materials, and it will allow patients to record notes and asthma triggers. The data will be available to the participants' providers from within their chart in Epic.

Data Collection. We will collect data on asthma-related healthcare utilization during the prior 12 months from our EHR and administrative sources. The asthma quality of life questionnaire will be administered through REDCap at baseline, 6-months, and 12-months post-randomization. Patient demographics, the patient activation measure (PAM-13), and the short literacy survey (SLS) will be administered at baseline only. We will also collect data on asthma-related healthcare utilization during the 12-month study period from the EHR and administrative sources.

Figure 1. Study Schedule

	Screen	Baseline	Midpoint	Endpoint

	Month 0	Month 0	Month 6	Month 12
**Administered with Patient**				
Inclusion/Exclusion Criteria	X			
Informed Consent	X			
Demographics	X			
Randomization		X		
Training on ASTHMA App		X*		
Mini AQLQ		X	X	X
PAM-13		X		
SLS		X		
**Collected from EHR and administrative sources**				
Asthma-related healthcare utilization	X			X

*For participants randomized to intervention group only

We will also conduct phone interviews a sample of patients and clinicians about their experience with the intervention using the same procedures as we used for design sessions in Phase I.

VI. BIOSTATISTICAL ANALYSIS

Phase I – Design Sessions

Interviews with clinicians and patients will be used to inform the design of the smartphone app and clinician dashboard. We will use NASSS, a novel framework specifically designed for developing and evaluating the scale and spread of technology-supported healthcare interventions. Consistent with our experience, NASSS emphasizes the importance of simplicity to scale and spread (e.g., interoperable technology, minimal training required, minimal changes in staff roles or workflows). We will therefore use this framework to systematically simplify as many dimensions as possible as we adapt and scale-up the practice model.

Phase II - RCT

RCT Statistical analysis plan

Overview:

For our primary analysis, we will test the hypothesis that asthma patients who use a clinically integrated asthma symptom monitoring smartphone application (intervention arm) will have greater improvement in asthma-related patient-reported quality of life over the course of the ~12-month study compared with patients who do not use the smartphone application (control arm). We will conduct secondary analyses of healthcare utilization, and exploratory analyses (see

below). We will use a generalized estimating equations (GEE) z-test (used for all types of data), which accounts for clustering by primary care provider (PCP).

Measures:

Primary outcome: The primary outcome will be change in the 15-item MiniAQLQ between baseline measure and final measure. We define each subject's baseline date as the date they completed the baseline MiniAQLQ. Subjects who were randomized but did not complete the baseline MiniAQLQ will be excluded from the primary / secondary analyses. Additionally, subjects who completed the baseline but not the final outcome measure will be excluded from the primary analysis. Thus, our primary analysis will only include participants who have a completed both the baseline and final outcome measure. The values of the MiniAQLQ range from 1 (worst quality of life) to 7 (highest quality of life). We will analyze MiniAQLQ as a continuous variable. We will administer this measure to all study participants (English and Spanish versions) at the beginning of the study, at 6 months, and at 12 months. This measure is validated; takes about 4 minutes to administer, and offers a comprehensive assessment of quality of life across four sub-domains: symptoms (5 items), activity limitations (4 items), emotional function (3 items), and exposure to environmental stimuli (3 items).[1] [2] The MiniAQLQ score is calculated as an average across these domains, and the minimum clinically important difference is 0.5.[3] In a prior mHealth study, mean MiniAQLQ scores increased from 4.3 to 4.8 post-intervention.[4]

Secondary outcome. The secondary outcome is non-routine asthma-related utilization, measured as the number of asthma-related emergency department (ED) visits, urgent care visits, and hospitalizations identified in the electronic health record (EHR) during the 12-month study period. From the EHR, we will include:

- Hospitalization: Primary or secondary encounter codes includes asthma
- ED: Primary or secondary encounter codes includes asthma
- UC: Primary or secondary encounter codes includes asthma

Exploratory outcomes: See exploratory analyses below.

Baseline demographics and characteristics of patient participants: From EHR: age, sex assigned at birth, race/ethnicity, marital status, primary language, education, socioeconomic status (median income by zip code), insurance status, clinic, PCP type, patient portal status, smoking status, visits to subspecialist 12 M before baseline, number of asthma medications at time of enrollment, presence or environmental or seasonal allergy or allergic rhinitis comorbidity based on problem list at time of baseline. From REDcap: phone type (e.g., iPhone, Android), baseline ACM score, baseline PAM score, baseline Health Literacy Score (SLS), study start date, time between completion of baseline and final surveys. We will also count

---

[1] Wilson SR, Rand CS, Cabana MD, Foggs MB, Halterman JS, Olson L, Vollmer WM, Wright RJ, Taggart V. Asthma outcomes: quality of life. Journal of Allergy and Clinical Immunology. 2012;129(3):S88-S123.
[2] Juniper E, Guyatt G, Cox F, Ferrie P, King D. Development and validation of the mini asthma quality of life questionnaire. European Respiratory Journal. 1999;14(1):32-8.
[3] Ibid. Juniper et al. (1999)
[4] Licskai CJ, Sands TW, Ferrone M. Development and pilot testing of a mobile health solution for asthma self-management: asthma action plan smartphone application pilot study. *Canadian respiratory journal.* 2013;20(4):301-6.

the number of unique PCP's with at least one patient in each arm and average number of patients per PCP in each arm.

Primary and secondary outcome analysis:

For our primary analysis, to estimate differences between intervention and control groups in our primary outcome (change in MiniAQLQ score) we will use robust linear regression models (generalized estimating equations [GEE]) with treatment arm as the only covariate, and clustering by PCP using GEE. Linear regression using GEE to compare mean scores between treatment and control groups is robust to non-normality of the outcomes. While we anticipate that baseline covariates (patient characteristics) will balance out across the two arms due to randomization, we may adjust for baseline characteristics that differ across arms for our primary and secondary analyses.

Baseline Data:

We will summarize all data using simple descriptive statistics (means with standard deviations for continuous variables, frequencies with percentages for categorical variables) overall and in each arm. We will then summarize baseline characteristics among 1) all enrolled patients in each group, 2) enrolled patients in each group who completed 12M surveys (for primary outcome only), and 3) enrolled patients in each group with missing 12M surveys (for primary outcome only).

Missing data:

As per Jakobsen et al.,[5] if there are less than 5% missing data, analysis of observed data (dropping subjects with missing data, also called a complete case analysis) will not lead to any bias. Also if there is > 5% missing data but are no significant differences across arms for distribution of follow up times (i.e., between baseline and completion of final primary outcome (MiniAQLQ)), the complete case analysis (in which we adjust for baseline covariates that differ b/w arms) is most appropriate along the guidelines given in Jakobsen et al. (2017). Furthermore, we will conduct a comparative analysis of the distribution of follow-up times (i.e., between baseline and completion of final primary outcome (MiniAQLQ) in the intervention and control arms. If we do not observe a significant difference across arms, then the complete case analysis is the most appropriate (with respect to power and bias). If there are significant differences ($p<0.05$) across arms for the distribution of follow up times, we will add follow-up time as a covariate the primary outcome. In essence, the treatment effect in the model for the primary outcome is adjusted for differences in follow-up times across groups. Furthermore, in that case, we will also calculate adjusted MiniAQLQ (primary outcome) means in the two groups, which will be adjusted to the average follow-up time across the two groups combined.

Secondary outcomes will be derived from the EHR – we will have all within-system EHR data for all patients in the study and therefore no missing data; secondary outcome data from outside health systems will not be included for the secondary outcomes analyses (see exploratory analyses), but we have no reason to believe such data will be different between intervention and

[5] Jakobsen, J.C., Gluud, C., Wetterslev, J. et al. When and how should multiple imputation be used for handling missing data in randomised clinical trials – a practical guide with flowcharts. BMC Med Res Methodol 17, 162 (2017). https://doi.org/10.1186/s12874-017-0442-1

control arms.

Power and sample size:

We computed our power calculation based on the original proposed number of clinics (originally proposed: 4 clinics; final number included in study: 7 clinics), the number of patients recruited (originally proposed: 500 patients; final number recruited and randomized: 413 patients), and the number of PCPs with at least one patient in the trial (originally proposed: 75; final number included in study: 104). We estimated a cluster size of 10 asthma patients per PCP.

For our primary outcome, asthma-related quality of life, we estimate mean MiniAQLQ scores of 4.3 at baseline. We conservatively estimate the intra-cluster (clinic) correlation coefficient (ICC) between MiniAQLQ scores from two patients with the same clinic to be approximately 0.01, commonly used for clustering of patients by PCP in this type of randomization study. We also assume that the SD for a patient's MiniAQLQ score is 1.0. Using a robust GEE z-test for ordinal categorical data (GEE does not assume normality of the outcome), and accounting for clustering of patients by PCP, we will have >90% power (alpha = 0.05) to detect a 0.5 point increase in the mean MiniAQLQ score (i.e., from 4.3 to 4.8 favoring the intervention). (Note: even though we expect the mean MiniAQLQ score to be approximately 4.3 in the control group, we will have >90% power to detect a 0.5 point decrease in mean MiniAQLQ score regardless of the mean MiniAQLQ score in the control group (i.e., from 5.1 to 5.6 favoring the intervention)).

For our secondary outcome, asthma-related healthcare utilization, we estimate a minimum of 10 ED visits (excluding hospitalizations and urgent care visits) per 100 asthma patients per year at baseline from national data sources.(1) Using a GEE Poisson z-test for rates (GEE is still unbiased even if the data are not Poisson) and accounting for clustering of patients by PCP (ICC = 0.01), we will have 80% power (alpha = 0.05) to detect a 50% decrease in the rate of ED visits per year (i.e., from 10 to 5 per 100 asthma patients per year).

Sensitivity analyses:

- Redo the primary analysis using imputation of missing 12M MiniAQLQ. Our imputation method will be to use the latest MiniAQLQ score completed, which might be the 12M, 6M or baseline.
- Redo the secondary outcome analysis with the subset of patients who completed the 12M MiniAQLQ.
- Redo the secondary outcome analysis including any utilization from external to the health system identified in the 6- and 12-month REDcap surveys.

Exploratory analyses:

- Redo the primary analysis for each of the 4 sub-domains of the MiniAQLQ as outcome measures: symptoms (5 items), activity limitations (4 items), emotional function (3 items), and exposure to environmental stimuli (3 items).
- Impact of intervention on outpatient utilization (visits and portal messages): As exploratory outcomes, we will compute the number of asthma-related outpatient visits within the MGB system as identified in the EHR during the study period. We will count any visit with an asthma-related billing code. We will compare results between the intervention and control. Separately, we will count the number of patient-generated portal messages sent by patients during the study period and compare results between the

intervention and control.

- Impact of intervention on prednisone prescriptions. We will test the hypothesis that the intervention group receives the same level or more prednisone prescriptions compared with the control group. We will calculate the 95% confidence interval for the difference. We will build GEE linear models with adjustment as described above for our primary analysis.
- Subgroup analyses: To explore how the effect of the intervention on the primary outcome varies within relevant population strata, we use regression models to assess the intervention's impact on the following variables:
    - Age at baseline: 18-44 vs 45-64 vs 65+ years
    - Sex: Male vs Female vs. Other
    - Race/ethnicity: Hispanic or Black vs. All other
    - Smoking status: Current vs. All other
    - Patient Activation Measure (PAM): High vs Low
    - Health Literacy (HL): High vs Low
    - Questionnaire completion percentage: High (>50%) vs Low
    - Retention: High (continued using app for 12 M) vs Low
- The subgroup analyses will be performed separately for each variable, and will fit models with interactions between treatment group and the subgroup to explore if the treatment effect is different across subgroups (a non-significant interaction implies that the treatment effect is similar across subgroups).
- Repeat measures analysis. For subjects with completed MiniAQLQ data at baseline, 6M, and 12M, we will compare change in MiniAQLQ baseline-6M, and 6M-12M for intervention and control arms using a differences-in-differences analysis. This will allow us to assess the degree to which any impact of the intervention occurred in the first 6M or the second 6M of the study period.

Definition of significance:

We will consider 2-sided P values to be significant at <0.05 for primary and secondary analyses.

Multiple hypothesis testing:

We will consider the intervention successful if the intervention arm shows a statistically significant improvement compared to the control arm on the primary outcome (improvement in MiniAQLQ over the course of the study period). Therefore, multiple testing is not needed. We do not plan to conduct multiple hypothesis testing for the subgroup analyses as their purpose is exploratory and for generating hypotheses.

VII. RISKS AND DISCOMFORTS

There are no known risks to participating in this study. Patient and clinician subjects may voluntarily participate and withdraw at any time. There is a theoretical risk of breach of data confidentiality and that PHI could become known to unauthorized persons, but we will take all steps necessary to protect PHI (see below). To mitigate this risk, we will follow all compliance and data confidentiality procedures for research at Partners. Specifically, we will use procedures consistent with sound research design and

which do not expose subjects to unnecessary risk. Data monitoring will be conducted regularly (see below). All data analyzed will be de-identified and reported in aggregate.

This is a minimal risk, quality improvement research study. There is no known physical or medical safety threat to patient or clinician subjects who participate this quality improvement research study. Furthermore, participation in this study is voluntary and all subjects will be informed that they may opt-out any time (see below). Given the minimal risk to subjects, our data safety and monitoring procedures, and the relatively small sample size, we do not plan on having drop criteria.

For patient and clinician subjects, there are no known risks to participating in our data collection activities. The main risks are breach of confidentiality and privacy of information shared. Every effort will be made to maintain confidentiality and privacy of information shared and collected and subsequent data analysis (see below), and efforts will be made to minimize the duration of data collection activities.

VIII. POTENTIAL BENEFITS

Patients may benefit by having the opportunity to discuss their experiences with asthma and contribution to research. Based on our previous pilot, ultimately, if successful, patients receiving the intervention may receive faster access to care, be more aware of their asthma symptoms, feel more connected to their clinician, and have better asthma control, possibly resulting in fewer hospitalizations and emergency room visits.

IX. MONITORING AND QUALITY ASSURANCE

The principal investigator and research assistant/project coordinator will monitor data collection activities, maintain data integrity and quality control, protect the rights, safety, and welfare of study subjects, and adhere to standards set by the Partners IRB. All data will be stripped of PHI and electronic data will be kept on a secured shared file area (SFA) behind the Partners firewall with anti-virus software or Partners Research Computing approved cloud storage services. Data about consented patients will also be stored behind the RAND Corporation firewall in accordance with the terms of the consent. Any paper forms and/or audio recordings will be destroyed after being transcribed into electronic format. Monitoring will be performed on a monthly basis during the study period and will include a review of enrollment, data analysis, and breaches of confidentiality, and any adverse events that may occur. Any adverse events will be graded as to their attribution to the intervention and reported according to IRB guidelines. The principal investigator will review any complaints reported from patient and clinician subjects. These will be reported to the Partners IRB annually or immediately if the complaint is serious. Given the minimal risk of the study, we are not planning to use automatic stopping rules or a DSMB. We emphasize that as a quality improvement intervention, this study does not involve any invasive procedures.

Voluntary participation in data collection activities are not expected to cause adverse events to patient or clinician subjects. However, if an adverse event occurs, the principal investigator will follow PHS HRC guidelines for adverse event reporting.

The principal investigator and research assistant/project coordinator will monitor and assure the validity and integrity of the data collection and adherence to the procedures outlined in this protocol. They will review the study database on a monthly basis, ensuring data integrity, accuracy, and completeness. The principal investigator will supervise research assistants/project coordinators in all data collection activities over the course of the study.

X. REFERENCES

1. Braido F. Failure in asthma control: reasons and consequences. *Scientifica*. 2013;2013.
2. Wang T, Srebotnjak T, Brownell J, Hsia RY. Emergency department charges for asthma-related outpatient visits by insurance status. *Journal of Health Care for the poor and underserved*. 2014;25(1):396.
3. Centers for Disease Control and Prevention. CDC Vital Signs: Asthma in the US. http://www.cdc.gov/vitalsigns/asthma/.
4. Gibson P, Powell H. Written action plans for asthma: an evidence-based review of the key components. *Thorax*. 2004;59(2):94-99.
5. Kessler KR. Relationship Between the Use of Asthma Action Plans and Asthma Exacerbations in Children With Asthma A Systematic Review. *Journal of Asthma & Allergy Educators*. 2011;2(1):11-21.
6. Lang DM. New asthma guidelines emphasize control, regular monitoring. *Cleveland Clinic journal of medicine*. 2008;75(9):641-653.
7. van der Meer V, van Stel HF, Bakker MJ, et al. Weekly self-monitoring and treatment adjustment benefit patients with partly controlled and uncontrolled asthma: an analysis of the SMASHING study. *Respiratory research*. 2010;11(1):74.
8. Wechsler ME. Getting Control of Uncontrolled Asthma. *The American journal of medicine*. 2014.
9. Van Gaalen JL, Beerthuizen T, van der Meer V, et al. Long-Term Outcomes of Internet-Based Self-Management Support in Adults With Asthma: Randomized Controlled Trial. *Journal of medical Internet research*. 2013;15(9).
10. Okelo SO, Butz AM, Sharma R, et al. Interventions to modify health care provider adherence to asthma guidelines: a systematic review. *Pediatrics*. 2013:peds. 2013-0779.
11. Wisnivesky JP, Lorenzo J, Lyn-Cook R, et al. Barriers to adherence to asthma management guidelines among inner-city primary care providers. *Annals of Allergy, Asthma & Immunology*. 2008;101(3):264-270.
12. Rudin RS, Fanta CH, Predmore Z, et al. Core Components for a Clinically Integrated mHealth App for Asthma Symptom Monitoring. *Appl Clin Inform*. Oct 2017;8(4):1031-1043.
13. Wilson SR, Rand CS, Cabana MD, et al. Asthma outcomes: quality of life. *Journal of Allergy and Clinical Immunology*. 2012;129(3):S88-S123.
14. Juniper E, Guyatt G, Cox F, Ferrie P, King D. Development and validation of the mini asthma quality of life questionnaire. *European Respiratory Journal*. 1999;14(1):32-38.
15. Licskai CJ, Sands TW, Ferrone M. Development and pilot testing of a mobile health solution for asthma self-management: asthma action plan smartphone application pilot study. *Canadian respiratory journal*. 2013;20(4):301-306.
16. Anselm S, Corbin J. *Basics of qualitative research: Techniques and procedures for developing grounded theory*. Thousand Oaks, CA: Sage Publications; 1998.
17. Silverman D. *Doing qualitative research: A practical handbook*: SAGE Publications Limited; 2013.
18. Cresswell J. *A Concise Introduction to Mixed Methods Research*. Los Angeles, CA: Sage Publications; 2015.
