# Supplementary material for: Between-Visit Asthma Symptom Monitoring With a Scalable Digital Intervention: A Randomized Clinical Trial
Source: JAMA Netw Open. 2025 Apr 23;8(4):e256219. doi: 10.1001/jamanetworkopen.2025.6219 (PMC12019512; doi:10.1001/jamanetworkopen.2025.6219)
Supplement: Supplement 2. — eAppendix 1. Intervention Components eAppendix 2. Survey Instruments Administered eTable 1. Baseline Patient Characteristics Stratified by Final Survey Completion Status eTable 2. Patients per Primary Care Physician Stratified by Patient Final Survey Completion Status eTable 3. Primary Analysis With Imputation of Missing 12-Month MiniAQLQ (Sensitivity Analysis) eTable 4. Secondary Analysis With Only Patients Who Completed 12-Month MiniAQLQ (Sensitivity Analysis) eTable 5. Secondary Outcome Analysis of Patient-Reported Utilization Internal and External to Health System (Sensitivity Analysis) eTable 6. Subgroup Analysis eTable 7. Patient Responses to Subdomains of the MiniAQLQ by Treatment Group (Exploratory) eTable 8. Outpatient Utilization by Treatment Group (Exploratory) eTable 9. Prednisone Prescriptions by Treatment Group (Exploratory) eTable 10. Repeat Measures Analyses (Exploratory) eTable 11. Adherence and Retention for Intervention Group Patients [file jamanetwopen-e256219-s002.pdf]

Supplemental Online Content

Rudin RS, Plombon S, Flores JS, et al. Between-visit asthma symptom monitoring with a scalable digital intervention: a randomized clinical trial. *JAMA Netw Open*. 2025;8(4):e256219. doi:10.1001/jamanetworkopen.2025.6219

eAppendix 1. Intervention Components

eAppendix 2. Survey Instruments Administered

eTable 1. Baseline Patient Characteristics Stratified by Final Survey Completion Status

eTable 2. Patients per Primary Care Physician Stratified by Patient Final Survey Completion Status

eTable 3. Primary Analysis With Imputation of Missing 12-Month MiniAQLQ (Sensitivity Analysis)

eTable 4. Secondary Analysis With Only Patients Who Completed 12-Month MiniAQLQ (Sensitivity Analysis)

eTable 5. Secondary Outcome Analysis of Patient-Reported Utilization Internal and External to Health System (Sensitivity Analysis)

eTable 6. Subgroup Analysis

eTable 7. Patient Responses to Subdomains of the MiniAQLQ by Treatment Group (Exploratory)

eTable 8. Outpatient Utilization by Treatment Group (Exploratory)

eTable 9. Prednisone Prescriptions by Treatment Group (Exploratory)

eTable 10. Repeat Measures Analyses (Exploratory)

eTable 11. Adherence and Retention for Intervention Group Patients

This supplemental material has been provided by the authors to give readers additional information about their work.

© 2025 Rudin RS et al. *JAMA Network Open*.

eAppendix 1. Intervention Components

Patient-facing intervention components

The app was available in Spanish and English and downloadable for iOS and Android devices. The app asked patients to complete weekly symptom questionnaires; showed their data in graph form; allowed them to enter notes, triggers and peak flow values; and provided educational videos such as how to use a rescue inhaler. For the weekly questionnaires, patients were prompted to complete an initial 5-item baseline Asthma Control Measure (ACM) questionnaire followed by similar weekly PRO questionnaires. If the PROs reflected problematic symptoms (defined as a 3-point worsening in the ACM compared to their baseline or prior week's score, or severest symptom on any one question), the app asked the patient if they wanted to request a call from a nurse. If the patient agreed, the app would send an electronic health record (EHR) inbox message to a triage nurse in the patient's primary care clinic. Patients also received reminders in the app prior to scheduled PCP visits to bring their smartphone and discuss their asthma data with their clinician.

Clinician-facing intervention components

Patient-entered data was available to clinicians within the patient chart in the electronic health records after clicking on the "ASTHMA data" tab. Primary care providers received EHR inbox messages prior to scheduled visits with participating patients reminding them to view the data in the dashboard.

Example EHR inbox message to nurse

• Patient has requested a callback from a nurse due to their asthma (requested at [time, date]).
• Call the patient at (XXX) XXX-XXX or a number listed in the patient chart.
• Patient's weekly self-reports show asthma symptoms were SEVERE.
• Patient has also reported the following this past week: ED visit(s).
• Click the "ASTHMA data" tab in the patient chart/encounter to review these data.
• Follow the "Nurse Directed Protocol for Managing Escalation of Asthma Symptoms: [link]
• ASTHMA research study IRB Protocol #2018P002394. Send question/comments to BWHAsthmaPRO@partners.org

Example EHR inbox message to PCP prior to scheduled visit

You may want to discuss asthma with PATIENT NAME during their visit with you tomorrow.
Their most recent weekly asthma symptom questionnaire (completed via smartphone app) showed POORLY CONTROLLED asthma.
Please remind the patient to complete their weekly questionnaires in the app.
Click "ASTHMA data" tab in Epic to see the data (add once if haven't already: click Epic menu item "Activity" -> ASTHMA data).
[Send question/comments to BWHAsthmaPRO@partners.org]

© 2025 Rudin RS et al. *JAMA Network Open*.

eAppendix 2. Survey Instruments Administered

Baseline

- Asthma Control Measure (ACM)
- Patient Activation Measure (PAM)
- Short Health Literacy survey (SLS)
- Mini Asthma Quality of Life (MiniAQLQ)
- Healthcare utilization due to asthma (emergency visits, urgent care visits, hospitalizations) in prior 12 months

6 months

- Mini Asthma Quality of Life (MiniAQLQ)
- Healthcare utilization due to asthma (emergency visits, urgent care visits, hospitalizations) in prior 6 months

12 months

- Mini Asthma Quality of Life (MiniAQLQ)
- Healthcare utilization due to asthma (emergency visits, urgent care visits, hospitalizations) in prior 6 months

© 2025 Rudin RS et al. *JAMA Network Open*.

eTable 1. Baseline Patient Characteristics Stratified by Final Survey Completion Status

Patient characteristics	Enrolled patients (secondary analysis)		Patients who completed 12-month survey (primary analysis)		Patients who did not complete 12-month survey	
	Intervention Group (N=211)	Usual-Care Group (N=202)	Completed 12-month MiniAQLQ Intervention Group (N=176)	Completed 12-month MiniAQLQ Usual-Care Group (N=190)	Intervention group (n=35)	Control group (n=12)
**Age, mean (SD)**	51.9 (15.5)	52.6 (15.4)	52.4 (15.3)	52.1 (15.5)	49.4 (16.1)	60.2 (12.6)
**Sex female assigned at birth, No. (%)**	157 (74.4)	164 (81.2)	125 (71.0)	155 (81.6)	32 (91.4)	9 (75.0)
**Race, No. (%)**						
White Non-Hispanic	116 (55.0)	110 (54.5)	106 (60.2)	105 (55.3)	10 (28.6)	5 (41.7)
Black Non-Hispanic	37 (17.5)	45 (22.3)	28 (15.9)	42 (22.1)	9 (25.7)	3 (25.0)
Hispanic	42 (19.9)	35 (17.3)	29 (16.5)	32 (16.8)	13 (37.1)	3 (25.0)
Other/Missing [a]	16 (7.6)	12 (5.9)	13 (7.4)	11 (5.8)	3 (8.6)	1 (8.3)
**Marital Status, No. (%)**						
Partnered	98 (46.5)	76 (37.6)	86 (48.9)	72 (37.9)	12 (34.3)	4 (33.3)
Single	111 (52.6)	125 (61.9)	88 (50.0)	117 (61.6)	19 (54.3)	4 (33.3)
Missing	2 (1.0)	1 (0.5)	2 (1.1)	1 (0.5)	4 (11.4)	4 (33.3)
**Primary language English, No. (%)**	202 (95.7)	192 (95.0)	169 (96.0)	183 (96.3)	33 (94.3)	9 (75.0)
**Education, No. (%)**						
8th grade or some high school but did not graduate	14 (6.6)	13 (6.4)	10 (5.7)	10 (5.3)	4 (11.4)	3 (25.0)
Graduated high school or GED	56 (26.5)	62 (30.7)	40 (22.7)	58 (30.5)	16 (45.7)	4 (33.3)
Graduated college	118 (55.9)	102 (50.5)	109 (61.9)	97 (51.1)	9 (25.7)	5 (41.7)
Missing	23 (10.9)	25 (12.4)	17 (9.7)	25 (13.2)	6 (17.1)	0 (0.0)
**Socioeconomic status (median income by zip code), No. (%)**						

© 2025 Rudin RS et al. *JAMA Network Open*.

Less than or equal to $76,585	54 (25.6)	52 (25.7)	39 (22.2)	51 (26.8)	15 (42.9)	1 (8.3)
$76,586 to $108,824	50 (23.7)	53 (26.2)	42 (23.9)	49 (25.8)	8 (22.9)	4 (33.3)
$108,825 to $137,102	53 (25.1)	49 (24.3)	48 (27.3)	44 (23.2)	5 (14.3)	5 (41.7)
Greater than $137,102	54 (25.6)	48 (23.8)	47 (26.7)	46 (24.2)	7 (20.0)	2 (16.7)
**Insurance status, No. (%)**						
Commercial	136 (64.5)	110 (54.5)	118 (67.1)	106 (55.8)	18 (51.4)	4 (33.3)
Medicaid	31 (14.7)	41 (20.3)	19 (10.8)	39 (20.5)	12 (34.3)	2 (16.7)
Medicare	43 (20.4)	50 (24.8)	38 (21.6)	44 (23.2)	5 (14.3)	6 (50.0)
Missing/Self-pay	1 (0.5)	1 (0.5)	1 (0.6)	1 (0.5)	0 (0.0)	0 (0.0)
**Employment status, No. (%)**						
Employed	132 (62.6%)	103 (51.0%)	112 (63.6%)	100 (52.6%)	20 (57.1%)	3 (25.0%)
Not Employed	34 (16.1%)	59 (29.2%)	26 (14.8%)	51 (26.8%)	8 (22.9%)	8 (66.7%)
Retired	28 (13.3%)	23 (11.4%)	24 (13.6%)	22 (11.6%)	4 (11.4%)	1 (8.3%)
Student	7 (3.3%)	7 (3.5%)	5 (2.8%)	7 (3.7%)	2 (5.7%)	0 (0.0%)
Missing	10 (4.7%)	10 (5.0%)	9 (5.1%)	10 (5.3%)	1 (2.9%)	0 (0.0%)
**Clinics, No. (%)**						
#1	18 (8.5)	15 (7.4)	11 (6.3)	14 (7.4)	6 (17.1)	1 (8.3)
#2	23 (10.9)	25 (12.4)	22 (12.5)	23 (12.1)	1 (2.9)	0 (0.0)
#3	41 (19.4)	38 (18.8)	36 (20.5)	34 (17.9)	5 (14.3)	4 (33.3)
#4	61 (28.9)	59 (29.2)	50 (28.4)	56 (29.5)	11 (31.4)	5 (41.7)
#5	28 (13.3)	24 (11.9)	21 (11.9)	22 (11.6)	7 (20.0)	2 (16.7)
#6	18 (8.5)	19 (9.4)	15 (8.5)	19 (10.0)	3 (8.6)	0 (0.0)
#7	22 (10.4)	22 (10.9)	21 (11.9)	22 (11.6)	1 (2.9)	0 (0.0)
Missing					1 (2.9)	
**PCP type, No. (%)[b]**						
Physician	206 (97.6)	198 (98.0)	173 (98.3)	186 (97.9)	33 (94.3)	12 (100.0)
Nurse Practitioner	5 (2.4)	2 (1.0)	3 (1.7)	2 (1.1)	2 (5.7)	0 (0.0)
Missing	--	2 (1.0)	--	2 (1.1)	--	--

© 2025 Rudin RS et al. *JAMA Network Open*.

**Patient Portal Status, No. (%)**						
Activated	202 (95.7)	191 (94.6)	171 (97.2)	180 (94.7)	31 (88.6)	11 (91.7)
Login Date within 6 months of study start	49 (23.2)	46 (22.8)	39 (22.2)	42 (22.1)	10 (28.6)	4 (33.3)
**Phone type, No. (%)**						
Android	47 (22.3)	54 (26.7)	40 (22.7)	51 (26.8)	7 (20.0)	3 (25.0)
iPhone	141 (66.8)	138 (68.3)	127 (72.2)	132 (69.5)	14 (40.0)	6 (50.0)
Other	4 (1.9)	0 (0.0)	4 (2.3)	0 (0.0)	0 (0.0)	0 (0.0)
Don’t know/ missing	19 (9.0)	10 (5.0)	5 (2.8)	7 (3.7)	14 (40.0)	3 (25.0)
**Asthma severity**						
**Smoking Status, No. (%)**						
Current smoker	9 (4.3)	15 (7.4)	8 (4.6)	14 (7.4)	1 (2.9)	1 (8.3)
Former smoker	62 (29.5)	55 (27.2)	48 (27.4)	52 (27.4)	14 (40.0)	3 (25.0)
Never smoker	139 (66.2)	132 (65.4)	119 (68.0)	124 (65.3)	20 (57.1)	8 (66.7)
Missing	1 (0.05)		1 (0.06)			
**Asthma exacerbation (one or more) in prior 12 months, No. patients (%)**	68 (32.2)	62 (30.7)	59 (33.5)	59 (31.1)	9 (25.7)	3 (25.0)
**Environmental, seasonal allergy, or allergic rhinitis comorbidity, No. (%)**	204 (96.7)	192 (95.1)	171 (97.2)	180 (94.7)	33 (94.3)	12 (100.0)
**General health**						
**Charlson comorbidity, Mean (SD)**	1.7 (1.4)	1.8 (1.6)	1.7 (1.4)	1.8 (1.7)	1.7 (1.4)	1.8 (1.1)
**BMI, Mean (SD)**	31.2 (8.0)	30.5 (7.7)	30.8 (8.0)	30.6 (7.8)	32.7 (7.6)	30.1 (6.0)
**ACM, Mean (SD)**	4.9 (4.0)	5.1 (3.8)	4.6 (4.0)	4.9 (3.7)	6.3 (3.9)	8.3 (3.8)
**PAM score level, No. (%)**						
1	8 (3.8)	10 (5.0)	7 (4.0)	9 (4.7)	1 (3.0)	1 (8.3)
2	18 (8.6)	18 (8.9)	10 (5.7)	15 (7.9)	8 (24.4)	3 (25.0)

© 2025 Rudin RS et al. *JAMA Network Open*.

3	67 (32.1)	56 (27.7)	60 (34.1)	53 (27.9)	7 (21.2)	3 (25.0)
4	116 (55.5)	118 (58.4)	99 (56.3)	113 (59.5)	17 (51.5)	5 (41.7)
Missing	2 (0.9)				2 (5.7)	
**Health Literacy (SLS), Mean (SD)**	13.8 (2.0)	13.7 (2.4)	14.0 (1.8)	13.8 (2.4)	12.8 (2.8)	12.1 (3.0)
**Questionnaires**						
**Time between baseline and 12-month MiniAQLQ, Mean No. days (SD)**	--	--	379.4 (36.9)	381.4 (34.8)	--	--
**Time between baseline and 12-month healthcare utilization, Mean No. days (SD)**	--	--	380.4 (38.6)	380.1 (36.6)	--	--
**MiniAQLQ**	5.18 (1.21)	5.04 (1.27)	5.34 (1.16)	5.08 (1.28)	4.33 (1.12)	4.44(0.91)

Abbreviations: SD, standard deviation; MiniAQLQ, Mini Asthma Quality of Life Questionnaire; SLS, Short Literacy Survey

[a] Other race includes American Indian or Alaska Native, Asian, Native Hawaiian or other Pacific Islander, or declined to answer

[b] Some records from one patient who withdrew from the study were lost resulting in one data point missing.

eTable 2. Patients per Primary Care Physician Stratified by Patient Final Survey Completion Status

Characteristic	Enrolled Intervention Group (N=211)	Enrolled Usual-Care Group (N=202)	Completed 12-month MiniAQLQ Intervention Group (N=176)	Completed 12-month MiniAQLQ Usual-Care Group (N=190)
No. PCPs with ≥ 1 patient	84	89	79	88
No. PCPs with > 1 patient (% of PCPs)	53 (63.1)	51 (57.3)	49 (62.0)	48 (54.5)
Mean No. patients per PCP (SD)	2.5 (1.7)	2.3 (1.7)	2.2 (1.4)	2.2 (1.6)

Abbreviations: PCP, primary care provider; SD, standard deviation

© 2025 Rudin RS et al. *JAMA Network Open*.

eTable 3. Primary Analysis With Imputation of Missing 12-Month MiniAQLQ (Sensitivity Analysis)*

MiniAQLQ [a]	Intervention Group (N=211)	Usual Care Group (N=202)	Difference Between Groups (95% CI)	P Value
Mean baseline score	5.20 (5.05, 5.35)	5.14 (4.99, 5.29)		
Mean 12-month score	5.57 (5.41, 5.72)	5.25 (5.09, 5.40)		
Change from baseline	0.37 (0.25, 0.49)	0.11 (-0.01, 0.23)	0.26 (0.09, 0.43)	<0.01

Abbreviations: CI, confidence interval; MiniAQLQ, Mini Asthma Quality of Life Questionnaire
*Values are clustered by primary care provider and adjusted for sex, income, insurance, employment, education, and timing of completion of the 12-month MiniAQLQ. We imputed values using baseline and 6-month MiniAQLQ, sex, income, insurance, employment, and education..

eTable 4. Secondary Analysis With Only Patients Who Completed 12-Month MiniAQLQ (Sensitivity Analysis)*

	Intervention Group (N=176)	Usual Care Group (N=190)	Difference (95% CI)	P Value
Mean number of non-routine asthma-related healthcare utilization events [a]	0.63	0.76	-0.13 (-0.42, 0.17)	0.39

Abbreviations: CI, confidence interval
*Values are clustered by primary care provider and adjusted for sex, income, insurance, employment and education.
[a] Measured as the mean number of asthma-related emergency department (ED) visits, urgent care visits, and hospitalizations (coded with asthma as a primary diagnosis) identified in the electronic health record (EHR) that occurred at MGB-affiliated institutions during the study period per patient.

© 2025 Rudin RS et al. *JAMA Network Open*.

eTable 5. Secondary Outcome Analysis of Patient-Reported Utilization Internal and External to Health System* (Sensitivity Analysis)

	Intervention Group (N=141)	Usual Care Group (N=146)	Difference (95% CI)	P Value
Mean total number of non-routine asthma-related healthcare utilization events during the study period per patient [a]	0.53	0.60	-0.08 (-0.42, 0.26)	0.66

Abbreviations: CI, confidence interval
*Values are clustered by primary care provider and adjusted for sex, income, insurance, employment and education.

[a] Measured as the mean total number of asthma-related healthcare utilization events (defined as the number of ED visits, urgent care visits, and hospitalizations) as self-reported by patients as part of the 6-month and 12-month questionnaires, including those internal and external to the health system. Only patients who have completed both the 6-month and 12-month questionnaires were included.

© 2025 Rudin RS et al. *JAMA Network Open*.

eTable 6. Subgroup Analysis*

Subgroup	Intervention Group (N=176)			Usual Care Group (N=190)			Difference (CI)	
**Overall**	**N**	**%**	**Change in MiniAQLQ [a], Mean**	**N**	**%**	**Change in MiniAQLQ [a], Mean**		
**Age at baseline**								
18-44	55	31.3	0.45	70	36.8	0.05	0.40 (0.13, 0.66)	
45-64	77	43.8	0.37	76	40.0	0.22	0.15 (-0.14, 0.44)	
65+ years	44	25.0	0.21	44	23.2	0.08	0.13 (-0.21, 0.48)	
**Sex**								
Male	51	29.0	0.44	35	18.4	0.10	0.34 (-0.14, 0.82)	
Female	125	71.0	0.33	155	81.6	0.13	0.20 (0.01, 0.38)	
**Race/ethnicity**								
Black or Hispanic	57	32.4	0.52	74	39.0	0.30	0.22 (-0.09, 0.53)	
Other	119	67.6	0.26	116	61.1	0.03	0.23 (0.01, 0.45)	
**Smoking status**								
Current	8	4.6	0.81	14	7.4	0.64	0.17 (-0.35, 0.69)	
Other	168	95.5	0.33	176	92.6	0.09	0.24 (0.05, 0.43)	
**Patient Activation Measure**								
High (levels 3, 4)	159	90.3	0.35	166	87.4	0.19	0.16 (-0.03, 0.34)	
Low	17	9.7	0.43	24	12.6	-0.34	0.77 (0.30, 1.24)	
**Health Literacy**								
High (14, 15)	137	77.8	0.33	146	76.8	0.12	0.21 (0.02, 0.41)	
Low (less than equal to 13)	39	22.2	0.43	44	23.2	0.15	0.28 (-0.16, 0.72)	
**Baseline MiniAQLQ**								
High (above median)	96	54.6	0.00	90	47.4	-0.18	0.18 (-0.02, 0.37)	
Low (below median)	80	45.5	0.76	100	52.6	0.42	0.33 (0.07, 0.59)	
**Baseline ACM**								
Controlled (0-2)	64	36.4	0.12	58	30.5	-0.03	0.15 (-0.05,0.35)	
Uncontrolled	112	63.6	0.49	132	69.5	0.19	0.30 (0.05, 0.54)	

Abbreviations: SD, standard deviation; CI, confidence interval; MiniAQLQ, Mini Asthma Quality of Life Questionnaire; ACM: asthma control measure

*Values are clustered by primary care provider and adjusted for sex, income, insurance, employment, education, and timing of completion of the 12-month MiniAQLQ.

[a] The MiniAQLQ range is 1 (worst) to 7 (best)

© 2025 Rudin RS et al. *JAMA Network Open*.

eTable 7. Patient Responses to Subdomains of the MiniAQLQ by Treatment Group (Exploratory)*

Subdomains of MiniAQLQ [a]	Intervention Group (N=176), Mean	Usual Care Group (N=190), Mean	Difference (95% CI)	
1. Symptoms				
Baseline MiniAQLQ	5.22	5.15		
12-month MiniAQLQ	5.60	5.29		
Change in MiniAQLQ	0.38	0.14	0.25 (0.04, 0.46)	
2. Activity limitations				
Baseline MiniAQLQ	5.85	5.74		
12-month MiniAQLQ	6.00	5.79		
Change in MiniAQLQ	0.15	0.05	0.10 (-0.11, 0.30)	
3. Emotional function				
Baseline MiniAQLQ	5.23	5.11		
12-month MiniAQLQ	5.68	5.26		
Change in MiniAQLQ	0.45	0.15	0.30 (0.03, 0.57)	
4. Exposure to environmental stimuli				
Baseline MiniAQLQ	4.69	4.74		
12-month MiniAQLQ	5.18	4.88		
Change in MiniAQLQ	0.48	0.14	0.35 (0.10, 0.59)	

Abbreviations: SD, standard deviation; CI, confidence interval; MiniAQLQ, Mini Asthma Quality of Life Questionnaire

*Values are clustered by primary care provider and adjusted for sex, income, insurance, employment, education, and timing of completion of the 12-month MiniAQLQ.

[a] The MiniAQLQ subdomains ranges are all 1 (worst) to 7 (best)

eTable 8. Outpatient Utilization by Treatment Group (Exploratory)*

	Intervention Group (N=211), Mean	Usual Care Group (N=202), Mean	Difference (95%CI)	
Number of asthma-related outpatient visits [a]	1.88	1.53	0.35 (-0.10, 0.80)	

Abbreviations: SD, standard deviation

*Values are clustered by primary care provider and adjusted for sex, income, insurance, employment, education, and timing of completion of the 12-month MiniAQLQ.

[a] Mean number of asthma-related outpatient visits within the health system as identified in the electronic health record (defined as outpatient visits at MGB-affiliated institutions, coded with asthma as a primary diagnosis) that occurred during the study period. Any visit with an asthma-related billing code was included.

© 2025 Rudin RS et al. *JAMA Network Open*.

eTable 9. Prednisone Prescriptions by Treatment Group (Exploratory)*

	Intervention Group (N=211), N (% of patienta)	Usual Care Group (N=202), N (% of patients)	Odds Ratio (95%CI)	
Number of patients with one or more prednisone prescriptions	37 (17.5%)	36 (17.8%)	1.03 (0.62, 1.69)	

Abbreviations: SD, standard deviation; CI, confidence interval
*Values are clustered by primary care provider and adjusted for sex, income, insurance, employment, education, and timing of completion of the 12-month MiniAQLQ.

eTable 10. Repeat Measures Analyses (Exploratory)*

	N	6-month – baseline MiniAQLQ [a], Mean (CI)	12-month – 6-month MiniAQLQ [a], Mean (CI)	12-month – baseline MiniAQLQ [a], Mean (CI)
Intervention Group	141	0.22 (0.10, 0.35)	0.05 (0.69) (-0.08, 0.18)	0.28 (0.15, 0.41)
Usual Care Group	146	-0.10 (-0.22, 0.03)	0.20 (0.07, 0.32)	0.09 (-0.04, 0.22)

Abbreviations: SD, standard deviation; CI, confidence interval; MiniAQLQ, Mini Asthma Quality of Life Questionnaire
*Values are clustered by primary care provider and unadjusted. Analysis only includes subjects who have completed MiniAQLQ at baseline, 6 months, and 12 months.
[a] The MiniAQLQ range is 1 (worst) to 7 (best)

eTable 11. Adherence and Retention for Intervention Group Patients*

	No. Patients	Mean baseline MiniAQLQ	Mean 12-month MiniAQLQ	12-month – baseline MiniAQLQ (CI)
High adherence	145	5.43	5.68	0.25 (0.13, 0.36)
Low adherence	31	5.16	5.84	0.68 (0.36, 1.00)
High retention	142	5.46	5.73	0.26 (0.14, 0.39)
Low retention	34	5.05	5.56	0.51 (0.23, 0.79)

Abbreviations: SD, standard deviation; CI, confidence interval; MiniAQLQ, Mini Asthma Quality of Life Questionnaire
*Values are clustered by primary care provider and adjusted for sex, income, insurance, employment, education, and timing of completion of the 12-month MiniAQLQ.
[a] The MiniAQLQ range is 1 (worst) to 7 (best)

© 2025 Rudin RS et al. *JAMA Network Open*.
