# Supplementary material for: Between-Visit Asthma Symptom Monitoring With a Scalable Digital Intervention: A Randomized Clinical Trial
Source: JAMA Netw Open. 2025 Apr 23;8(4):e256219. doi: 10.1001/jamanetworkopen.2025.6219 (PMC12019512; doi:10.1001/jamanetworkopen.2025.6219)
Supplement: Supplement 3. — Data Sharing Statement [file jamanetwopen-e256219-s003.pdf]

Data Sharing Statement

Rudin. Between-Visit Asthma Symptom Monitoring With a Scalable Digital Intervention. *JAMA Netw Open*. Published April 23, 2025. doi:10.1001/jamanetworkopen.2025.6219

Data
Additional Information: ClinicalTrials.gov Identifier: NCT04401332
https://clinicaltrials.gov/study/NCT04401332
Data available: Yes
Data types: Deidentified participant data, Data dictionary
How to access data: Requests for data should be sent to the corresponding author at rrudin@rand.org.
When available: With publication

Supporting Documents
Document types: None

Additional Information
Who can access the data: We will make the data available to researchers under reasonable requests whose proposed use has been approved.
Types of analyses: Data will be made available for research purposes only.
Mechanisms of data availability: Data will be made available after approval of a proposal.
